# Intelligent control decision integrating fuzziness and randomness for automatic management of cash flow

**DOI:** 10.1371/journal.pone.0292748

**Published:** 2024-03-01

**Authors:** Hongli Wang, Liguo Fei, Yuqiang Feng

**Affiliations:** 1 School of Economics and Trade, Fujian Jiangxia University, Fuzhou, Fujian, China; 2 School of Political Science and Public Administration, Shandong University, Qingdao, Shandong, China; 3 School of Economics and Management, Harbin Institute of Technology, Harbin, Heilongjiang, China; Vinnytsia National Technical University, UKRAINE

## Abstract

Automatic management of cash flow from the perspective of cybernetics decisions can improve work efficiency and accuracy of cash flow management. Disadvantage of traditional fuzzy control method is that it only expresses fuzziness and ignores randomness. The automatic management of cash flow involves variables representing the fuzziness and randomness of human cognition which need new calculation methods to solve. Based on fuzzy control this paper proposes a cloud set control decision method for cash flow management. Cloud set and its *I* operation and *P* operation are described. Methods are studied including observation variables and control variables, fuzziness of observation variables and control variables, description of rules, and cloud reasoning based on cloud set. The method is applied successfully in automatic management of cash flow in which control amount of expenditure intensity is -2.285. It is shown that this method can effectively obtain reasonable control quantities considering fuzzy and random properties by the comparison with fuzzy control method. The method for automatic management of cash flow proposed has greater objectivity and effectiveness for the integration of fuzzy and randomness representing human cognition and decision.

## Introduction

Fuzzy mathematics plays an important role in many uncertain information processing fields. In 1965 the fuzzy mathematics theory was proposed by Prof. Zadeh [1]. Methods based on fuzzy set provide useful mathematical tools which are applied in medical diagnosis [2], electronic [3], mobile robot [4], machine learning [5], etc. to deal with the problems with uncertainty information. The traditional fuzzy control is to map the exact value to a fuzzy value, and then use the fuzzy value to generate a new fuzzy value through fuzzy controller operation based on the fuzzy membership, and finally return the fuzzy value to generate the exact value through precision. Traditional fuzzy controller has some disadvantages such as inferior adaptability due to the invariable membership function parameters and too many subjective factors [6]. Fuzzy logic was proposed by Zadeh in 1988 [7]. The relevant methods and applications of fuzzy logic have been widely studied [8–10]. It is the core of traditional fuzzy control theory. But fuzzy control theory and method ignore randomness because fuzzy mathematics method only expresses fuzziness but not randomness. Fuzziness and randomness are the two most important uncertainties inherent in human cognition, which have attracted great attention in artificial intelligence research [11]. Cloud model suggests a novel approach for managing and representing the intrinsic uncertainty associated with linguistic concepts, particularly focusing on fuzziness, randomness, and their interrelationship. Cloud set theory and its method are derived from the cloud model, serving as an expansion and distillation of the cloud model in the set theory [12, 13]. It is reasonable to believe that the control method based on cloud set theory can better achieve the control of uncertain things with the attributes of fuzzy and randomness. Therefore, studying the control method based on cloud set and applying it to the intelligent control decision will be able to control well with fuzziness and randomness.

Cash flow refers to the inflow and outflow of cash in the operation of enterprises [14]. In a broad sense, cash includes cash on hand, bank deposits, and cash equivalents. Generating positive long-term cash flow is vital for a firm’s sustainability [15]. Cash flow plays an important role in the operation of an enterprise. If there is a problem with cash flow it will be difficult for the enterprise to continue to operate. There are many examples of bankruptcy caused by the problem of cash flow. Some data show that in developed countries many bankrupt companies are profit-making companies from the perspective of accounting, and it is not the account losses that lead to their bankruptcy but the lack of cash. This shows that cash flow determines the success or failure of an enterprise and cash flow control can create fundamental value for the enterprise. The company’s cash levels can vary considerably over time depending on payment and collection cycle [16]. It can be controlled to a certain extent. Cash flow control aims to keep the net cash (the difference between the total value of incoming cash and the total value of outcoming cash) not less than the safety threshold. The net cash safety threshold of different types of enterprises will be very different. However, it should be noted that when the net cash is far greater than the safety threshold, although it is safer, it is not the most cost-effective for the enterprise and may affect the efficiency of the enterprise. Intelligent control decision of cash flow can effectively avoid liquidity risk caused by capital shortage and effectively prevent financial risk. When the net cash volume is less than the safety threshold, the cash reserve should be strengthened to promote the fund recovery of accounts receivable, sales income cash, debt financing or stock financing, government subsidies, and other projects. When the net cash volume is greater than the safety threshold, the cash expenditure should be strengthened, such as expanding reproduction, investing abroad, purchasing goods, purchasing raw materials, etc., to improve the operating efficiency of the enterprise and promote the benign development of the enterprise. It can be seen that the process of cash inflow and outflow is a dynamic control process. Some forecasting models or methods are used to forecast and monitor cash flow [17–20]. Modelization, digitalization, and informationize are the inevitable trends of management of cash flow [21–23]. It can use the automatic control method of the automatic control subject for the automatic management of cash flow, especially the fuzzy control theory and method which are widely used in automatic control [24–27]. Relative studies on cash flow management include control of cash flow [28–30], prediction of cash flow [31–34], feasibility of cash flow [35], risk manaement of cash flow [36], valuation application of cash flow [37], optimization of cash flow [38], and deficit detection of cash flow [39]. All of these studies consider fuzziness but most of them do not consider randomness. A few of them consider randomness only as random-based searching mechanism for time series data [32] or stochastic fuzzy variables with normally distributed density functions [39]. Comparative analysis of relative studies can be seen in Table 1 in detail. Further, there is a great demand for intelligent control decision of cash flow. Some studies have indicated good potential for using artificial intelligence to reduce reliance on human input in cash flow control. At present there is little research on the automatic control method of cash flow. The automatic management of cash flow can focus on maintaining the safety threshold level of cash flow, and carry out dynamic regulation and control after the cash flow deviates from the safety threshold level. The content of regulation and control includes limiting or permitting cash reserves, expenditures, and the strength of reserves and expenditures. Our method based on normal cloud set for automatic control of in-out value of cash flow in this paper not only considers fuzziness and randomness but also organically integrates them.

**Table 1:** Comparative analysis of relative studies.

Scope of cash flow management	Method	Consider fuzziness?	Consider randomness?	References
Control	Artificial intelligence including K-means clustering, genetic algorithm (GA), fuzzy logic (FL), and neural network (NN)	Yes	No	[28]
	Evolutionary fuzzy hybrid neural network	Yes	No	[29]
	Fuzzy number and operations	Yes	No	[30]
Prediction	Time-dependent support vector machines	Yes	No	[31]
	Enhanced Time-Dependent Evolutionary Fuzzy Support Vector Machines	Yes	Yes (Only random-based searching mechanism for time series data)	[32]
	New interval type-2 fuzzy model	Yes	No	[33]
	Fuzzy modelling	Yes	No	[34]
Feasibility	MAIRR method under fuzzy conditions	Yes	No	[35]
Risk	Adaptive fuzzy controller	Yes	No	[36]
Valuation application	fuzzy discounted cash flow model	Yes	No	[37]
Optimization for maximization	Hybrid genetic algorithm	Yes	No	[38]
Deficit Detection	Mamdani fuzzy logic	Yes	Yes (Stochastic fuzzy variables with normally distributed density functions)	[39]
Automatic Control for in-out value of cash flow	Our method based on normal cloud set in this paper	Yes	Yes (Integrating fuzziness and randomness attributes)	-

The motivations of paper are as follows: (1) Providing useful intelligent control decision methods for dealing with the management of cash flow. With the integration of AI and cash flow management the intelligence is the general trend of cash flow management. Based on the improvement and application of existing intelligent control decision methods the purpose of this paper is to provide effective methods to adapt to management of cash flow. (2) Overcoming the shortcoming of lacking the ability to objectively express fuzziness and randomness in the existing intelligent control decision methods. The control of cash flow involves variables with fuzziness and randomness. In those existing intelligent control decision methods, the fuzziness is only considered and the randomness is ignored. This paper proposes a cash flow automatic control method based on normal cloud set which objectively expresses the fuzziness and randomness of uncertain concepts in cash flow control. (3) Proposing and verifying the effectiveness of normal cloud model and its intelligent control decision method in simulating human cognition in cash flow management. The intelligent control decision method for cash flow management based on the normal cloud model is originated. It is verified through specific case application of cash flow management and comparison analysis.

The scientific innovation of this paper is that an intelligent control decision-making method integrating fuzziness and randomness is originally constructed for the automatic management of cash flow. The addition of randomness makes the expression of uncertainty more objective and reasonable. Compared with previous researches this method effectively simulates the uncertainty of human decision-making cognition based on cloud set. In practice, it provides an effective method for intelligent decision-making problems with uncertainty such as cash flow management.

The sections of this paper are arranged as follows. First, the relevant research results, motivation, and innovation of this study are described. Second, the article reviews the *I* operation and *P* operation of cloud sets and cloud sets as preliminaries. Third, the intelligent control decision method based on normal cloud sets is given, including the automatic control principle and the automatic control method which solves the key problems such as the deterministic observation and control quantity based on cloud sets, the fuzziness of input and output quantities, and cloud reasoning based on cloud sets. Fourth, the intelligent control decision method of cash flow is given to manage automatically the cash flow. The comparison analysis with fuzzy intelligent control is given to verify the feasibility and effectiveness. Finally, the conclusions and prospects are given.

## Preliminaries

### Cloud set for simulation of human decision

The theory and method of set theory are the foundation of various branches of modern mathematics and many fields of science and technology. It is the basic tool to express the basic field of mathematics. Set theory is the basis of mathematics, computer science, intelligence science, and other disciplines [40]. Cloud model founded by Academician Li of Chinese Academy of Sciences is a model that can effectively represent the uncertainty of event called “fuzzy-randomness” [11]. The cloud model uses hyper-entropy to reflect the cloud shape and represents the "thickness" of the cloud which associates the fuzziness with the randomness representing the uncertainty of human cognition [41]. Wang created the basic set theory and method of cloud model from the perspective of set theory which is called cloud set. Its constituent elements are based on cloud model. He proposed the *I* operation and *P* operation methods of cloud set. On this basis, he gave the basic operation of cloud set [12] and studied the cloud relationship equation and cloud relationship matrix [13]. Cloud set effectively simulates the uncertainty of human cognition of things integrating fuzziness and randomness. The definitions of cloud set and cloud set element are provided in the following:

**Definition 1** [12]. **Cloud set**. Let *U* be a universe. The subset of *U* is composed of some elements in *U*. The subset $\underset{\approx }{A}=\{{\overset{\approx }{x}}_{1},{\overset{\approx }{x}}_{2},\cdots ,{\overset{\approx }{x}}_{n}\}$ is called as cloud set when the membership degree of element $\overset{\approx }{x}$ of subset is the set which is represented based on the cloud model *C*(*Ex*, *En*, *He*). The element membership function $\overset{\approx }{x}$ is recorded as $u_{\underset{\approx }{A}}(\overset{\approx }{x})$. The subset is called normal cloud set in which *Ex*, *En* and *He* is respectively expectation, entropy, and hyper-entropy if *C*(*Ex*, *En*, *He*) is the normal cloud model.

**Definition 2** [12]**. Cloud set element.** Cloud set element is defined as the number with random membership in cloud set. The *x* is recorded as $\overset{\approx }{x}$ if it is the element of cloud set. It is different from fuzzy set that $u_{\underset{\approx }{A}}(\overset{\approx }{x})$ is the random number but not the certain number in [0,1]. In the normal cloud set the membership of which is *C*_*j*_(*Ex*_*j*_, *En*_*j*_, *He*_*j*_) the generating method of random membership $u_{\underset{\approx }{A}}(\overset{\approx }{x})$ of element $\overset{\approx }{x}$ is as follows:

**Step 1**. Generating random number $En_{j}^{\prime }$ with *En*_*j*_ as the expectation and *He*_*j*_ as the standard deviation;

**Step 2.** Generating normal random number ${Ex}_{j}^{\prime }$ with *Ex*_*j*_ as the expectation and $En_{j}^{\prime }$ as the standard deviation. Let $u_{\underset{\approx }{A}}(\overset{\approx }{x})=e^{-\frac{(\overset{\approx }{x}-Ex{)}^{2}}{2E{n_{j}^{\prime }}^{2}}}$ be the random membership of cloud set element $\overset{\approx }{x}$.

Among them, because the generated random number in step 1 has multiple possible values in step 2 the random membership $u_{\underset{\approx }{A}}(\overset{\approx }{x})$ has multiple possible values.

### *I* Operation and *P* operation in normal cloud set

The *I* operation of normal cloud set is to calculate the value range of random membership degree. The calculation method is denoted in the following Formula (1) [12]:

$$I(u_{\underset{\approx }{A}}(\overset{\approx }{x}))=[e^{-\frac{(\overset{\approx }{x}-Ex_{\underset{\approx }{A}}{)}^{2}}{2(En_{\underset{\approx }{A}}-3He_{\underset{\approx }{A}}{)}^{2}}},\;e^{-\frac{(\overset{\approx }{x}-Ex_{\underset{\approx }{A}}{)}^{2}}{2(En_{\underset{\approx }{A}}+3He_{\underset{\approx }{A}}{)}^{2}}}],\forall \overset{\approx }{x}\in \underset{\approx }{A}$$ (1)

where $I(u_{\underset{\approx }{A}}(\overset{\approx }{x}))$ is the interval of random membership degree $u_{\underset{\approx }{A}}(\overset{\approx }{x})$ of cloud set elements $\overset{\approx }{x}$. Formula (1) is mainly based on the 3*σ* principle of normal distribution which points out that the element value distribution: 99.73% probability falls within the limit interval (*μ*−3*σ*, *μ*+3*σ*) where *σ* is the standard deviation and *μ* is the mean value [42]. According to this principle it is approximately considered the probability of value distribution in this interval (*μ*−3*σ*, *μ*+3*σ*) is 1.

The possibility that one interval is greater than another interval is calculated by the *P* operation. It is recorded as Formula (2):

$$P(I_{1}\geq I_{2})=P(I(u_{\underset{\approx }{A}}(\overset{\approx }{x}))\geq I(u_{\underset{\approx }{B}}(\overset{\approx }{x}))$$ (2)

where *P*(*I*_1_≥*I*_2_) represents the possibility that interval $I(u_{\underset{\approx }{A}}(\overset{\approx }{x}))$ is greater than or equal to interval $I(u_{\underset{\approx }{B}}(\overset{\approx }{x}))$. The detail calculation method is denoted in Formula (3) [12]:

$$P(I(u_{\underset{\approx }{B}}(\overset{\approx }{x})\geq I(u_{\underset{\approx }{B}}(\overset{\approx }{x}))=P([a^{l},b^{r}]\geq [a^{l},b^{r}])$$

$$=\left\{ \begin{array}{cc} 1 & a^{l}\geq b^{r}\\ \frac{a^{r}-b^{r}}{a^{r}-b^{l}}+\frac{b^{r}-a^{l}}{2(a^{r}-b^{l})} & b^{l}<a^{l}<b^{r}<a^{r}\\ \frac{1}{2} & a^{l}=b^{l},a^{r}=b^{r}\\ \frac{a^{r}-b^{l}}{2(b^{r}-a^{l})} & a^{l}<b^{l}<a^{r}<b^{r}\\ \frac{a^{r}-b^{r}}{a^{r}-a^{l}}+\frac{b^{r}-b^{l}}{2(a^{r}-a^{l})} & a^{l}<b^{l}<b^{r}<a^{r}\\ \frac{a^{r}-a^{l}}{2(b^{r}-b^{l})} & b^{l}<a^{l}<a^{r}<b^{r}\\ 0 & b^{l}\geq a^{r} \end{array}\right.$$ (3)

where $I(u_{\underset{\approx }{A}}(\overset{\approx }{x}))$ is represented using the interval [*a*^*l*^, *a*^*r*^] and $I(u_{\underset{\approx }{B}}(\overset{\approx }{x}))$ is represented using the interval [*b*^*l*^, *b*^*r*^].

## Key method of intelligent control decision system based on normal cloud set

### Fuzziness of observation variables and control variables

**Definition 3.** Define the core membership curve of cloud set as the curve the hyper-entropy *He* of which is zero. If cloud set is $\underset{\approx }{A}=C_{j}(Ex_{j},En_{j},He_{j})$ the core membership curve of normal cloud set is denoted in Formula (4):

$$u_{\underset{\approx }{A}}(\overset{\approx }{x})=e^{-\frac{(\overset{\approx }{x}-Ex_{j}{)}^{2}}{2{{En}_{j}}^{2}}}$$ (4)

Normal cloud subset is used to express the observation variables and control variables. Their digital characters including expectation *Ex*, entropy *En*, and hyper-entropy *He* are calculated by the following method.

(1) Supposing the universe of cloud subset is {−n,−n+1,…,0,…,n−1,n}. The number of words of language set is *m* that is generally an odd number not less than 3. According to *n* and *m* every normal cloud subset’s position of center is determined as the expectation *Ex* (the leftmost and rightmost cloud subset is respectively right-semi cloud and left semi-cloud) in Formula (5):

$$\left\{{Ex}_{-\frac{m-1}{2}},{Ex}_{-\frac{m-1}{2}+1},\ldots ,0,{Ex}_{\frac{m-1}{2}-1},{Ex}_{\frac{m-1}{2}}\}=\{-n,-\frac{\frac{m-3}{2}*2n}{m-1},\ldots ,-\frac{2*2n}{m-1},-\frac{1*2n}{m-1},0,\frac{1*2n}{m-1},\frac{2*2n}{m-1},\ldots ,\frac{\frac{m-3}{2}*2n}{m-1},n\right\}$$ (5)

The abscissa value of intersection *X*_*in*_ of core membership function of two adjacent cloud subsets are denoted as follows in Formula (6):

$$\left\{-\frac{(m-2)*n}{m-1},\ldots ,-\frac{3*n}{m-1},-\frac{1*n}{m-1},\frac{1*n}{m-1},\frac{3*n}{m-1},\ldots ,\frac{(m-2)*n}{m-1}\right\}$$ (6)

(2) The cloud subset is evenly distributed. It is objective and reasonable that the membership degree of abscissa value of intersection *X*_*in*_ is set as 0.5 because there can bring good execution performance which is commonly used in the related fuzzy control research in the references [43–45]. When the *Ex* and *X*_*in*_ have already gained the *En* can be resolved by the following Formula (7):

$$e^{-\frac{(X_{in}-Ex{)}^{2}}{2{En}^{2}}}=0.5\Rightarrow En=\frac{\left|X_{in}-Ex\right)}{\sqrt{-2ln0.5}}$$ (7)

(3) When the *Ex*, *X*_*in*_, and *En* have already gained the hyper-entropy *He* can be to resolved by the following Formula (8) in which the equal sign is satisfied:

$$e^{-\frac{(X_{in}-Ex{)}^{2}}{2{\left(En+3He\right)}^{2}}-}e^{-\frac{(X_{in}-Ex{)}^{2}}{2{\left(En-3He\right)}^{2}}}\leq \delta$$ (8)

where δ is the given threshold value which represents the random variation range of membership degree caused by hyper-entropy at the intersection of adjacent cloud subsets.

If the actual variation range is [*a*, *b*], then observation variables and control variables are converted into several levels of the cloud subset universe according to the following Formula (9) [46]:

$$y=\frac{2n}{b-a}(x-\frac{a+b}{2})$$ (9)

where *n* is the number of divisions. Based on this, the mapping factor method can be directly used to map the actual deviation *d*, the actual deviation change *dc*, and actual control *u* to deviation variable *D*, deviation change variable *DC*, and control variable *U* in intelligent control system using following Formulas (10–12) [46].

$$D=k_{d}d$$ (10)

$$DC=k_{c}dc$$ (11)

$$U=k_{u}u$$ (12)

where *k*_*d*_, *k*_*c*_, and *k*_*u*_ are mapping factors which can be converted into quantification factors and scale factors through certain correspondence conversions.

### Cloud relationship and cloud rule composition

The following uses the synthesis method of cloud reasoning, that is, the cloud implication relations of all rules are comprehensively processed to obtain the total cloud relations of the whole rule base. Among them, the cloud operations within the rules take the intersection set, and the cloud operations between the rules take the union set, which has high operation efficiency.

If there are *n* rules in the rule base, their cloud implication relations are *R*_1_, *R*_2_,…,*R*_*n*_, then for each cloud relationship *R*_*i*_ and for regulation $A_{1}\;is\;\underset{\approx }{A_{i1}}\;and\;A_{2}\;is\;\underset{\approx }{A_{i2}}\ldots A_{j}\;is\;\underset{\approx }{A_{ij}}\to B\;is\;\underset{\approx }{B_{i}}$ is denoted as follows in Formula (13):

$$R_{A_{1}\;is\;\underset{\approx }{A_{i1}}\;and\;A_{2}\;is\;\underset{\approx }{A_{2i}}\ldots A_{j}\;is\;\underset{\approx }{A_{ji}}\to B\;is\;\underset{\approx }{B_{i}}}=R_{i}=\underset{\approx }{A_{i1}}\cap \underset{\approx }{A_{i2}}\cap \ldots \cap \underset{\approx }{A_{ij}}\cap \underset{\approx }{B_{i}}$$ (13)

It can be expressed as a cloud subset on *A*_*i*_∩*B*_*i*_. The random membership degree $u_{R_{i}=A_{i}\cap B_{i}}$ is calculated as follows in Formula (14):

$$u_{R_{i}={R_{i}=\underset{\approx }{A_{i1}}\cap \underset{\approx }{A_{i2}}\cap \ldots \cap \underset{\approx }{A_{ij}}\cap \underset{\approx }{B_{i}}}_{R_{i}=\underset{\approx }{A_{i1}}\cap \underset{\approx }{A_{i2}}\cap \ldots \cap \underset{\approx }{A_{ij}}\cap \underset{\approx }{B_{i}}}}=u_{\underset{\approx }{A_{i1}}}(\overset{\approx }{x})\wedge u_{\underset{\approx }{A_{i2}}}(\overset{\approx }{x})\wedge \ldots \wedge {u_{\underset{\approx }{A_{ij}}}(\overset{\approx }{x})\wedge u}_{\underset{\approx }{B_{i}}}(\overset{\approx }{x})$$ (14)

where the symbol "∧" indicates the operation of "taking the smallest”, that is, any $u_{\underset{\approx }{A}}(\overset{\approx }{x})\subseteq [0,1]$ and $u_{\underset{\approx }{B}}(\overset{\approx }{x})\subseteq [0,1]$, "∧" operation of cloud set is defined as based on *I* operation and *P* operation of cloud number in Formula (15) [12]:

$$u_{\underset{\approx }{A}}(\overset{\approx }{x})\wedge u_{\underset{\approx }{B}}(\overset{\approx }{x})=\left\{ \begin{array}{cc} u_{\underset{\approx }{A}}(\overset{\approx }{x}) & \frac{P(I(u_{\underset{\approx }{A}}(\overset{\approx }{x}))>I(u_{\underset{\approx }{B}}(\overset{\approx }{x})))}{P(I(u_{\underset{\approx }{B}}(\overset{\approx }{x}))>I(u_{\underset{\approx }{A}}(\overset{\approx }{x})))}<1\\ u_{\underset{\approx }{A}}(\overset{\approx }{x}),u_{\underset{\approx }{B}}(\overset{\approx }{x}) & \frac{P(I(u_{\underset{\approx }{A}}(\overset{\approx }{x}))>I(u_{\underset{\approx }{B}}(\overset{\approx }{x})))}{P(I(u_{\underset{\approx }{B}}(\overset{\approx }{x}))>I(u_{\underset{\approx }{A}}(\overset{\approx }{x})))}=1\\ u_{\underset{\approx }{B}}(\overset{\approx }{x}) & \frac{P(I(u_{\underset{\approx }{A}}(\overset{\approx }{x}))>I(u_{\underset{\approx }{B}}(\overset{\approx }{x})))}{P(I(u_{\underset{\approx }{B}}(\overset{\approx }{x}))>I(u_{\underset{\approx }{A}}(\overset{\approx }{x})))}>1\\ u_{\underset{\approx }{B}}(\overset{\approx }{x}) & P(I(u_{\underset{\approx }{B}}(\overset{\approx }{x}))>I(u_{\underset{\approx }{A}}(\overset{\approx }{x})))=0 \end{array}\right.$$ (15)

The cloud set rule of the system is a synthesis of multiple statements. It can be expressed as a cloud subset on $\underset{\approx }{A_{1}}\cup \underset{\approx }{A_{2}}\cup \ldots \cup \underset{\approx }{A_{n}}$, that is, cloud relation:

$$R=R_{1}\cup R_{2}\cup \ldots R_{n}.$$ (16)

The corresponding random membership degree is calculated as follows:

$${u_{R}=u}_{R_{1}\cup R_{2}\cup \ldots R_{n}}=u_{\underset{\approx }{R_{1}}}(\overset{\approx }{x})\vee u_{\underset{\approx }{R_{2}}}(\overset{\approx }{x})\ldots \vee u_{\underset{\approx }{R_{n}}}(\overset{\approx }{x}).$$ (17)

where the symbol "∨" indicates the operation of "taking the largest”, that is, for any $u_{\underset{\approx }{A}}(\overset{\approx }{x})\subseteq [0,1]$ and $u_{\underset{\approx }{B}}(\overset{\approx }{x})\subseteq [0,1]$, "∨" operation of cloud set is defined as follows based on *I* operation and *P* operation of cloud number in Formula (18) [12]:

$$u_{\underset{\approx }{A}}(\overset{\approx }{x})\vee u_{\underset{\approx }{B}}(\overset{\approx }{x})=\left\{ \begin{array}{cc} u_{\underset{\approx }{A}}(\overset{\approx }{x}) & \frac{P(I(u_{\underset{\approx }{A}}(\overset{\approx }{x}))>I(u_{\underset{\approx }{B}}(\overset{\approx }{x})))}{P(I(u_{\underset{\approx }{B}}(\overset{\approx }{x}))>I(u_{\underset{\approx }{A}}(\overset{\approx }{x})))}>1\\ u_{\underset{\approx }{A}}(\overset{\approx }{x}),u_{\underset{\approx }{B}}(\overset{\approx }{x}) & \frac{P(I(u_{\underset{\approx }{A}}(\overset{\approx }{x}))>I(u_{\underset{\approx }{B}}(\overset{\approx }{x})))}{P(I(u_{\underset{\approx }{B}}(\overset{\approx }{x}))>I(u_{\underset{\approx }{A}}(\overset{\approx }{x})))}=1\\ u_{\underset{\approx }{B}}(\overset{\approx }{x}) & \frac{P(I(u_{\underset{\approx }{A}}(\overset{\approx }{x}))>I(u_{\underset{\approx }{B}}(\overset{\approx }{x})))}{P(I(u_{\underset{\approx }{B}}(\overset{\approx }{x}))>I(u_{\underset{\approx }{A}}(\overset{\approx }{x})))}<1\\ u_{\underset{\approx }{A}}(\overset{\approx }{x}) & P(I(u_{\underset{\approx }{B}}(\overset{\approx }{x}))>I(u_{\underset{\approx }{A}}(\overset{\approx }{x})))=0 \end{array}\right.$$ (18)

### Clarity of control variable *U*

#### Ideal center of gravity method

The so-called ideal center of gravity method is to preliminary determine that the final result is a positive domain, then take the non-positive domain value in the domain as the minimum value of the membership interval, and take the positive domain value in the domain as the maximum value of the membership interval, and vice versa.

Supposing the universe of cloud subset is the level {*-n*, *-n+*1, *…*, 0, *…*, *n-*1, *n*} and their corresponding results of approximate reasoning are as follows:

$$\left[[{Le}_{-n},{Ri}_{-n}][{Le}_{-n+1},{Ri}_{-n+1}]\ldots [{Le}_{0},{Ri}_{0}]\ldots [{Le}_{n-1},{Ri}_{n-1}][{Le}_{n},{Ri}_{n}]\right]$$ (19)

The ideal center of gravity method to calculate the result of clarity of control variable *U* is in the following:

(1) If the result is predicted as a positive domain the result of clarity of control variable *U* is as follows in Formula (20):

$${ouptput}_{U}=\frac{\left({Le}_{-n}*(-n)+{Le}_{-n+1}*(-n+1)+\ldots +{Le}_{0}*0+\ldots +{Ri}_{n-1}*(n-1)+{Ri}_{n}*n\right)}{\left({Le}_{-n}+{Le}_{-n+1}+\ldots +{Le}_{0}+{Ri}_{n-1}+{Ri}_{n}\right)}.$$ (20)

(2) If the result is predicted as a negative domain the result of clarity of control variable *U* is as follows in Formula (21):

$${ouptput}_{U}=\frac{\left({Ri}_{-n}*(-n)+{Ri}_{-n+1}*(-n+1)+\ldots +{Ri}_{0}*0+\ldots +{Le}_{n-1}*(n-1)+{Le}_{n}*n\right)}{\left({Ri}_{-n}+{Ri}_{-n+1}+\ldots +{Ri}_{0}+{Le}_{n-1}+{Le}_{n}\right)}$$ (21)

#### Other methods

Other methods include about the all-maximum-membership degree, the one-maximum-membership degree, and the mean of interval of membership degree. In the all-maximum-membership degree right endpoints of all intervals of membership degree are selected as the membership degree of output:

$${ouptput}_{U}=\frac{\left({Ri}_{-n}*(-n)+{Ri}_{-n+1}*(-n+1)+\ldots +{Ri}_{0}*0+\ldots +{Ri}_{n-1}*(n-1)+{Ri}_{n}*n\right)}{({Ri}_{-n}+{Ri}_{-n+1}+\ldots +{Ri}_{0}+{Ri}_{n-1}+{Ri}_{n}).}$$ (22)

In one-maximum-membership degree method the maximum one in $[{Le}_{-n},{Ri}_{-n}],$ $[{Le}_{-n+1},{Ri}_{-n+1}],$ …, $[{Le}_{0},{Ri}_{0}],$ …, $[{Le}_{n-1},{Ri}_{n-1}],$ and [*Le*_*n*_, *Ri*_*n*_] is firstly selected, then its’ one corresponding term in the universe {−n,−n+1,…,0,…,n−1,n} is found. The product of the two ones is the final output value. Calculating the result when the right endpoint of interval of membership degree is selected as its value.

In the mean of interval of membership degree method, the mean values are used as the value of interval of membership degree.


$${ouptput}_{U}=\frac{\frac{{(Le}_{-n}+{Ri}_{-n})(-n)}{2}+\frac{{{(Le}_{-n+1}+Ri}_{-n+1})(-n+1)}{2}+\ldots +\frac{{{(Le}_{0}+Ri}_{0})*0}{2}+\ldots +\frac{{(Le}_{n-1}+{Ri}_{n-1})(n-1)}{2}+\frac{({Le}_{n}+{Ri}_{n})n}{2}}{\frac{{Le}_{-n}+{Ri}_{-n}}{2}+\frac{{{Le}_{-n+1}+Ri}_{-n+1}}{2}+\ldots +\frac{{{Le}_{0}+Ri}_{0}}{2}+\ldots +\frac{{Le}_{n-1}+{Ri}_{n-1}}{2}+\frac{{Le}_{n}+{Ri}_{n}}{2}}$$ (23)

## Cash flow intelligent control decision system based on normal cloud set

### Basic principle of automatic control of the company’s cash flow

There is a cash pool in the company. Funds enter the cash pool due to reserves and flow out of the cash pool due to expenditures. Design an intelligent control decision to control the cash flow to be stable near the safety level point along with the reserve and expenditure of funds. According to the management experience of the company’s cash flow, the following basic control rules can be obtained:

(1) When the deviation abbreviated as *D* is negative and large if the deviation changes abbreviated as *DC* is negative then the deviation tends to increase. To eliminate the existing negative deviation as soon as possible and suppress the deviation from becoming large the quantity of cash flow abbreviated as *U* is taken as negative large.

When the deviation *D* is negative and large and the deviation change *DC* is positive the system itself tends to reduce the deviation. In order to eliminate the deviation as soon as possible without causing an overshoot the quantity of cash flow *U* should be negative and small.

(2) When the deviation *D* is negative and small the system is close to the steady state. If the deviation change *DC* is negative selecting the quantity of cash flow as negative middle to suppress the deviation *D* from changing in the negative direction. If the deviation change *DC* is positive the system has a tendency to eliminate the negative small deviation. Selecting the quantity of cash flow *U* as zero or positive small.
(3) When the deviation *D* is positive the control idea is the same as (1) and (2) except that the signs are opposite.

**Fig 1:**
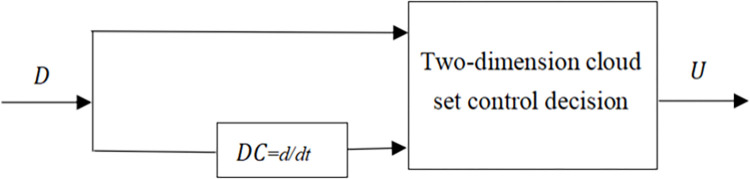
Company cash flow intelligent control decision.

The two-dimensional intelligent control decision is the most frequently used in control fields. According to the above experience, the two-dimension intelligent control decision for controlling of cash flow is designed as follows in Fig 1.

### Automatic control decision method of company cash flow

#### Determine observation variables and control variables

Define the cash amount at the safety level point as *m*_0_, and the actual cash amount held by the company is *m*. Select the cash deviation *D* as follows:

$$D=\Delta m=m_{0}-m.$$ (24)

The deviation of the current cash amount from the safety point is taken as an observation variable *D*. Define the change in *D* per unit time as *DC*. Define changes in the strength of adjusting capital reserves or expenditures as the control variables *U*.

#### Fuzziness and randomness of observation variables and control variables

In the following, in addition to the original levels data of observation variables and control variables, the subsequent data are derived or calculated based on the levels data.

*(1) observation variables*. The actual variation range of the deviation *D* is hypothetically converted into seven levels of the cloud subset universe: -3, -2, -1, 0, +1, +2, +3.

The deviation *D* is divided into five cloud sets. When the ordinate of the intersection between the core membership of cloud sets is set to 0.5 and the length threshold of the random membership at the intersection is set to *δ* = 0.3, according to the Formulas (5–8), the parameters are calculated in Formula (25):

$$\left\{ \begin{array}{c} \left\{{Ex}_{NB},{Ex}_{NS},{Ex}_{ZO},{Ex}_{PS},{Ex}_{PB}\}=\{-3,-\frac{\frac{5-3}{2}*2*3}{5-1},0,\frac{\frac{5-3}{2}*2*3}{5-1},3\}=\{-3,-1.5,0,1.5,3\right\}\\ \left\{X_{in}\}=\{-\frac{3*3}{5-1},-\frac{1*3}{5-1},\frac{1*3}{5-1},\frac{3*3}{5-1}\}=\{-2.25,-0.75,0.75,2.25\right\}\\ \left\{{En}_{NB},{En}_{NS},{En}_{ZO},{En}_{PS},{En}_{PB}\}=\{\frac{\left|-2.25-(-3)\right)}{\sqrt{-2ln0.5}},\frac{\left|-0.75-(-1.5)\right)}{\sqrt{-2ln0.5}},\frac{\left|0.75-0\right)}{\sqrt{-2ln0.5}},\frac{\left|0.75-1.5\right)}{\sqrt{-2ln0.5}},\frac{\left|2.25-3\right)}{\sqrt{-2ln0.5}}\}=\{0.637,0.637,0.637,0.637,0.637\right\}\\ e^{-\frac{(X_{in}-Ex{)}^{2}}{2{\left(En+3He\right)}^{2}}-}e^{-\frac{(X_{in}-Ex{)}^{2}}{2{\left(En-3He\right)}^{2}}}=0.3\Rightarrow \{{He}_{NB},{He}_{NS},{He}_{ZO},{He}_{PS},{He}_{PB}\}=\{0.046,0.046,0.046,0.046,0.046\} \end{array}\right.$$ (25)

**Fig 2:**
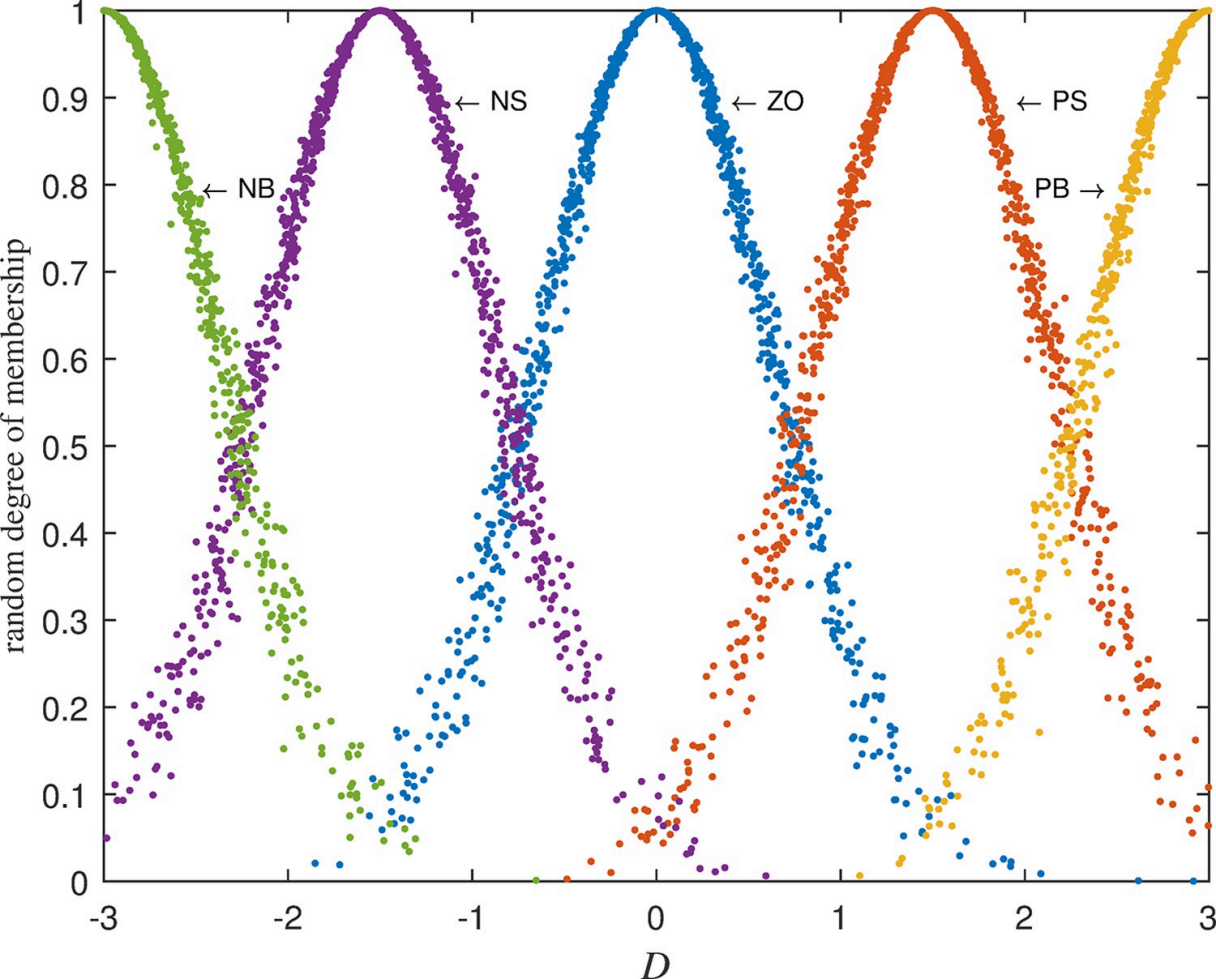
Cloud set division of deviation *D*.

The five cloud sets for deviation *D* are obtained as follows: Negative Big (NB)~C(−3,0.637,0.046), Negative Small(NS)~C(−1.5,0.637,0.046), Zero(ZO)~C(0,0.637,0.046), Positive small(PS)~C(1.5,0.637,0.046), Positive Big(PB)~C(3,0.637,0.046). Their diagrams of cloud set are shown in Fig 2 below:

The seven grades of the deviation *D* are brought into its five cloud sets and the value of the random membership interval is obtained based on the *I* operation in Formula (1) of Cloud sets. Taking Negative Big (NB)~C(−3,0.637,0.046) as an example the seven levels of deviation *D* are calculated as follows in Formula (26):

$${I(u}_{\underset{\approx }{NB}}(\overset{\approx }{x}=-3))=[e^{-\frac{(-3-(-3){)}^{2}}{{2(0.637-3*0.046)}^{2}}},e^{-\frac{(-3-(-3){)}^{2}}{{2(0.637+3*0.046)}^{2}}}]=[1,1]$$ (26)

Similarly in Formulas (27–32):

$${I(u}_{\underset{\approx }{NB}}(\overset{\approx }{x}=-2))=[e^{-\frac{(-2-(-3){)}^{2}}{{2(0.637-3*0.046)}^{2}}},e^{-\frac{(-2-(-3){)}^{2}}{{2(0.637+3*0.046)}^{2}}}]=[0.134,0.435]$$ (27)

$${I(u}_{\underset{\approx }{NB}}(\overset{\approx }{x}=-1))=[0.0003,0.0358]=[0,0.036]$$ (28)

$${I(u}_{\underset{\approx }{NB}}(\overset{\approx }{x}=0))=[0.0001*10^{-4},0.0005]=[0,0.001]$$ (29)

$${I(u}_{\underset{\approx }{NB}}(\overset{\approx }{x}=1))=[0.0001*10^{-10},0.0001*10^{-2}]=[0,0]$$ (30)

$${I(u}_{\underset{\approx }{NB}}(\overset{\approx }{x}=2))=[0.0001*10^{-18},0.0009*10^{-6}]=[0,0]$$ (31)

$${I(u}_{\underset{\approx }{NB}}(\overset{\approx }{x}=3))=[0.0004*10^{-28},0.0009*10^{-10}]=[0,0]$$ (32)

**Fig 3:**
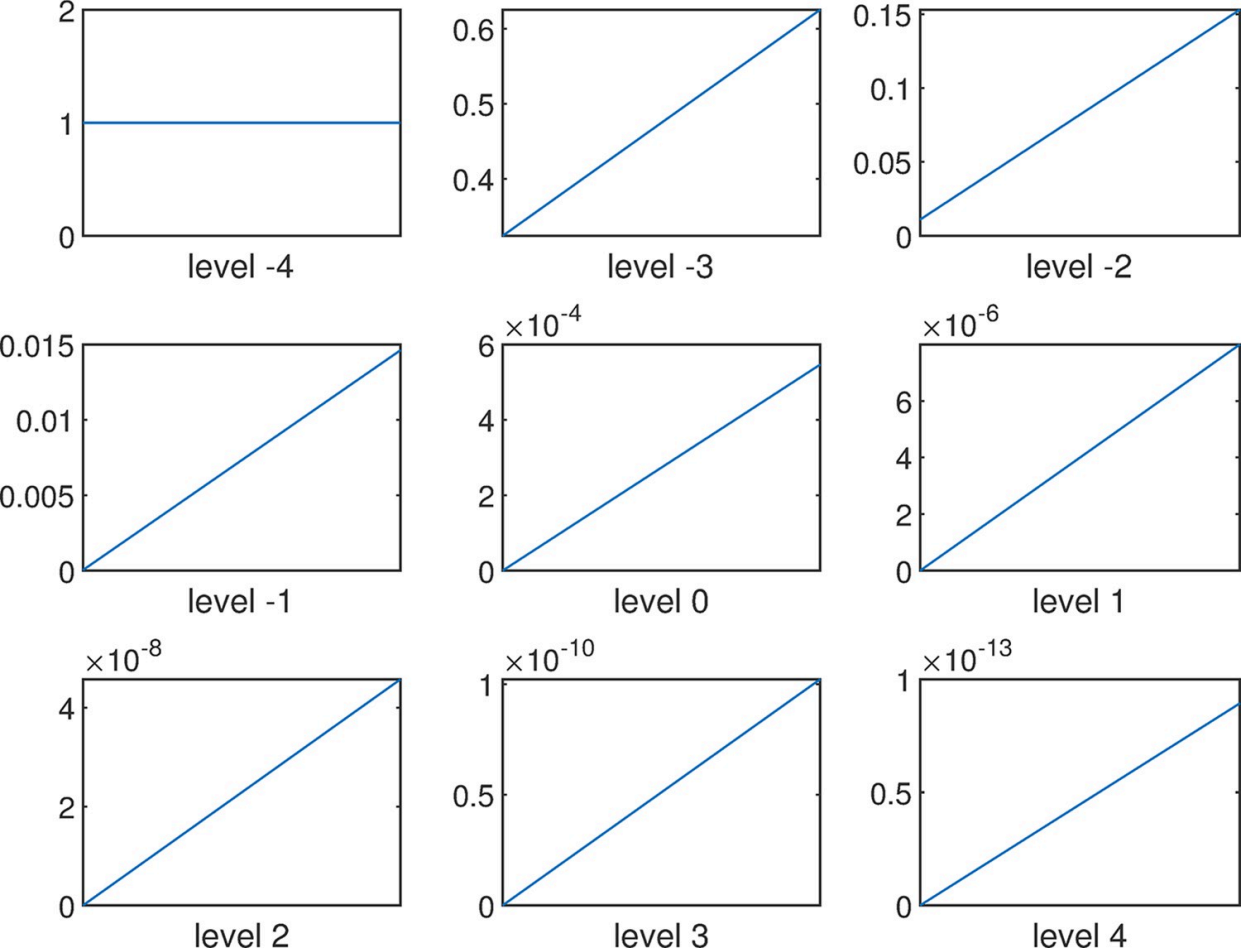
Random membership of NB cloud set of deviation *D*.

The above random membership degrees are shown in Fig 3:

The above process is repeated to calculate the value of the random membership interval of Negative Small(NS)~C(−1.5,0.637,0.046), Zero(ZO)~C(0,0.637,0.046), Positive Small(PS)~C(1.5,0.637,0.046), Positive Big(PB)~C(3,0.637,0.046). The following random membership interval assignment in Table 2 is obtained:

**Table 2:** Random membership interval assignment table of deviation *D*.

Degree of membership		Level						
		-3	-2	-1	0	1	2	3
**Cloud set**	**NB**	[1.000,1.000]	[0.134,0.435]	[0.000,0.036]	[0.000,0.001]	[0.000,0.000]	[0.000,0.000]	[0.000,0.000]
	**NS**	[0.011,0.154]	[0.605,0.812]	[0.605,0.812]	[0.011,0.154]	[0.000, 0.006]	[0.000,0.000]	[0.000,0.000]
	**ZO**	[0.000,0.001]	[0.000,0.036]	[0.134,0.435]	[1.000,1.000]	[0.134,0.435]	[0.000,0.036]	[0.000,0.001]
	**PS**	[0.000,0.000]	[0.000,0.000]	[0.000,0.006]	[0.011,0.154]	[0.605,0.812]	[0.605,0.812]	[0.011,0.154]
	**PB**	[0.000,0.000]	[0.000,0.000]	[0.000,0.000]	[0.000,0.001]	[0.000,0.036]	[0.134,0.435]	[1.000,1.000]

Similarly, the variation *DC* of deviation *D* is divided into seven grades -3, -2, -1, 0, +1, +2, +3 and five cloud subsets Negative Big (NB)~C(−3,0.637,0.046), Negative Small(NS)~C(−1.5,0.637,0.046), Zero(ZO)~C(0,0.637,0.046), Positive Small(PS)~C(1.5,0.637,0.046), Positive Big(PB)~C(3,0.637,0.046). It has the same random membership interval as the deviation *D* and the assignment table is the same as Table 2.

**Fig 4:**
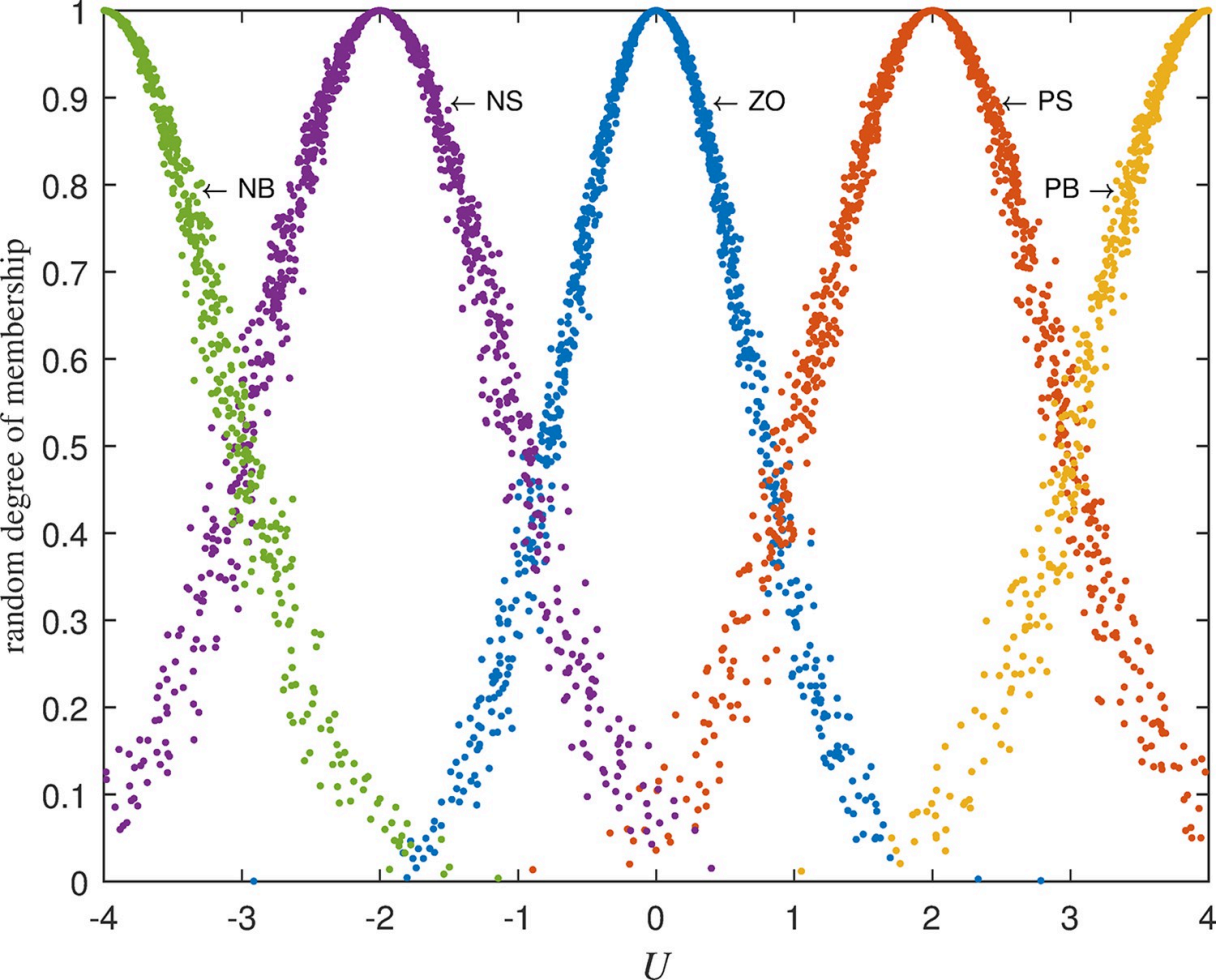
Cloud set division of control variable *U*.

*(2) Control variables*. The control variable *U* is the change of the strength of adjusting the cash reserve or expenditure. According to the same method as deviation *D*, control variable *U* is divided into five fuzzy sets: Negative Big(NB)~C(−4,0.849,0.061), Negative Small(NS)~C(−2,0.849,0.061), Zero(ZO)~C(0,0.849,0.061), Positive Small(PS)~C(2,0.849,0.061), Positive Big(PB)~C(4,0.849,0.061). And the variation range of *U* is divided into nine levels: -4, -3, -2, -1, 0, +1, +2, +3, +4. Cloud set division of the obtained control quantity *U* is shown in Fig 4.

Using the same method as the calculation of deviation *D* the following random membership interval assignment for control variable *U* in Table 3 is obtained:

**Table 3:** Random membership interval assignment table of control variable *U*.

degree of membership		Level								
		-4	-3	-2	-1	0	1	2	3	4
cloud set	**NB**	[1.000,1.000]	[0.324,0.625]	[0.011,0.153]	[0.000,0.015]	[0.000,0.001]	[0.000,0.000]	[0.000,0.000]	[0.000,0.000]	[0.000,0.000]
	**NS**	[0.011,0.153]	[0.324,0.625]	[1.000,1.000]	[0.324,0.625]	[0.011,0.153]	[0.000,0.015]	[0.000,0.001]	[0.000,0.000]	[0.000,0.000]
	**ZO**	[0.000,0.001]	[0.000,0.015]	[0.011,0.153]	[0.324,0.625]	[1.000,1.000]	[0.324,0.625]	[0.011,0.153]	[0.000,0.015]	[0.000,0.001]
	**PS**	[0.000,0.000]	[0.000,0.000]	[0.000,0.001]	[0.000,0.015]	[0.011,0.153]	[0.324,0.625]	[1.000,1.000]	[0.324,0.625]	[0.011,0.153]
	**PB**	[0.000,0.000]	[0.000,0.000]	[0.000,0.000]	[0.000,0.000]	[0.000,0.001]	[0.000,0.015]	[0.011,0.153]	[0.324,0.625]	[1.000,1.000]

### Description of rules based on cloud set

According to the experience of company executives and financial experts the following cloud rules are designed in Table 4 containing 25 cloud rules.

**Table 4:** Cloud rules in intelligent control decision of cash flow.

*U*		*D*				
		NB	NS	ZO	PS	PB
***DC***	**NB**	PB	PB	PS	PS	ZO
	**NS**	PB	PS	PS	ZO	NS
	**ZO**	PS	PS	ZO	NS	NS
	**PS**	PS	ZO	NS	NS	NB
	**PB**	ZO	NS	NS	NB	NB

### Cloud reasoning based on cloud set

According to the cloud relationship and cloud rule composition method, for the rule NB_*D*_∩NB_*DC*_→PB_*U*_, having the following calculation in Formula (33):

$$u_{R_{{NB}_{D}\cap {NB}_{DC}\to {PB}_{U}}}=u_{\underset{\approx }{{{NB}_{D}}_{i}}}(\overset{\approx }{x})\wedge u_{\underset{\approx }{{{NB}_{DC}}_{i}}}(\overset{\approx }{x})\wedge u_{\underset{\approx }{{PB}_{U}}}(\overset{\approx }{x})$$ (33)

where $u_{\underset{\approx }{{{NB}_{D}}_{i}}}(\overset{℘}{x})\wedge u_{\underset{\approx }{{{NB}_{DC}}_{i}}}(\overset{\approx }{x})$ is calculated in Formula (34):


$$u_{\underset{\approx }{{{NB}_{D}}_{i}}}(\overset{℘}{x})\wedge u_{\underset{\approx }{{{NB}_{DC}}_{i}}}(\overset{\approx }{x})=[\begin{array}{c} \left[1.000,1.000\right]\\ \left[0.134,0.435\right]\\ \left[0.000,0.036\right]\\ \left[0.000,0.001\right]\\ \left[0.000,0.000\right]\\ \left[0.000,0.000\right]\\ \left[0.000,0.000\right] \end{array}][[1.000,1.000][0.134,0.435][0.000,0.036][0.000,0.001][0.000,0.000][0.000,0.000][0.000,0.000]]=\left[\begin{array}{c} \left[1.000,1.000][0.134,0.435][0.000,0.036][0.000,0.001][0.000,0.000][0.000,0.000][0.000,0.000\right]\\ \left[0.134,0.435][0.134,0.435][0.000,0.036][0.000,0.001][0.000,0.000][0.000,0.000][0.000,0.000\right]\\ \left[0.000,0.036][0.000,0.036][0.000,0.036][0.000,0.001][0.000,0.000][0.000,0.000][0.000,0.000\right]\\ 0.000,0.001][0.000,0.001][0.000,0.001][0.000,0.001][0.000,0.000][0.000,0.000][0.000,0.000]\\ \left[0.000,0.000][0.000,0.000][0.000,0.000][0.000,0.000][0.000,0.000][0.000,0.000][0.000,0.000\right]\\ \left[0.000,0.000][0.000,0.000][0.000,0.000][0.000,0.000][0.000,0.000][0.000,0.000][0.000,0.000\right]\\ \left[0.000,0.000][0.000,0.000][0.000,0.000][0.000,0.000][0.000,0.000][0.000,0.000][0.000,0.000\right] \end{array}\right]$$ (34)

In the above calculation $u_{\underset{\approx }{{{NB}_{D}}_{i}}}(\overset{\approx }{x})$ is the membership degree vector which is the membership degree of observation variable *D* belonging to NB. $u_{\underset{\approx }{{{NB}_{DC}}_{i}}}(\overset{\approx }{x})$ is the membership degree vector which is the membership degree of observation variable *DC* belonging to NB. Here every membership degree in vector is an interval. The resulting matrix of above calculation is converted into the column vector to perform the following calculation by bringing into the following Formula (35):

$$u_{\underset{\approx }{{{NB}_{D}}_{i}}}(\overset{\approx }{x})\wedge u_{\underset{\approx }{{{NB}_{DC}}_{i}}}(\overset{\approx }{x})\wedge u_{\underset{\approx }{{PB}_{U}}}(\overset{\approx }{x})=$$

$$\left[\begin{array}{c} \left[1.000,1.000\right]\\ \left[0.134,0.435\right]\\ \left[0.000,0.036\right]\\ \left[0.000,0.001\right]\\ \left[0.000,0.000\right]\\ \left[0.000,0.000\right]\\ \left[0.000,0.000\right]\\ \left[0.134,0.435\right]\\ \left[0.134,0.435\right]\\ \left[0.000,0.036\right]\\ \left[0.000,0.001\right]\\ \left[0.000,0.000\right]\\ \left[0.000,0.000\right]\\ \left[0.000,0.000\right]\\ \left[0.000,0.036\right]\\ \left[0.000,0.036\right]\\ \left[0.000,0.036\right]\\ \left[0.000,0.001\right]\\ \left[0.000,0.000\right]\\ \left[0.000,0.000\right]\\ \left[0.000,0.000\right]\\ 0.000,0.001]\\ \left[0.000,0.001\right]\\ \left[0.000,0.001\right]\\ \left[0.000,0.001\right]\\ \left[0.000,0.000\right]\\ \left[0.000,0.000\right]\\ \left[0.000,0.000\right]\\ \left[0.000,0.000\right]\\ \left[0.000,0.000\right]\\ \left[0.000,0.000\right]\\ \left[0.000,0.000\right]\\ \left[0.000,0.000\right]\\ \left[0.000,0.000\right]\\ \left[0.000,0.000\right]\\ \left[0.000,0.000\right]\\ \left[0.000,0.000\right]\\ \left[0.000,0.000\right]\\ \left[0.000,0.000\right]\\ \left[0.000,0.000\right]\\ \left[0.000,0.000\right]\\ \left[0.000,0.000\right]\\ \left[0.000,0.000\right]\\ \left[0.000,0.000\right]\\ \left[0.000,0.000\right]\\ \left[0.000,0.000\right]\\ \left[0.000,0.000\right]\\ \left[0.000,0.000\right]\\ \left[0.000,0.000\right] \end{array}\right]{\left[\begin{array}{c} \left[0.000,0.000\right]\\ \left[0.000,0.000\right]\\ \left[0.000,0.000\right]\\ \left[0.000,0.000\right]\\ {}[0.000,0.001\\ \left[0.000,0.015\right]\\ \left[0.011,0.153\right]\\ \left[0.324,0.625\right]\\ \left[1.000,1.000\right] \end{array}\right]}^{T}=\left[\begin{array}{c} \left[0.000,0.000][0.000,0.000][0.000,0.000][0.000,0.000][0.000,0.001][0.000,0.015][0.011,0.153][0.324,0.625][1.000,1.000\right]\\ \left[0.000,0.000][0.000,0.000][0.000,0.000][0.000,0.000][0.000,0.001][0.000,0.015][0.011,0.153][0.134,0.435][0.134,0.435\right]\\ \left[0.000,0.000][0.000,0.000][0.000,0.000][0.000,0.000][0.000,0.001][0.000,0.015][0.000,0.036][0.000,0.036][0.000,0.036\right]\\ \left[0.000,0.000][0.000,0.000][0.000,0.000][0.000,0.000][0.000,0.001][0.000,0.001][0.000,0.001][0.000,0.001][0.000,0.001\right]\\ \left[0.000,0.000][0.000,0.000][0.000,0.000][0.000,0.000][0.000,0.000][0.000,0.000][0.000,0.000][0.000,0.000][0.000,0.000\right]\\ \left[0.000,0.000][0.000,0.000][0.000,0.000][0.000,0.000][0.000,0.000][0.000,0.000][0.000,0.000][0.000,0.000][0.000,0.000\right]\\ \left[0.000,0.000][0.000,0.000][0.000,0.000][0.000,0.000][0.000,0.000][0.000,0.000][0.000,0.000][0.000,0.000][0.000,0.000\right]\\ \left[0.000,0.000][0.000,0.000][0.000,0.000][0.000,0.000][0.000,0.001][0.000,0.015][0.011,0.153][0.134,0.435][0.134,0.435\right]\\ \left[0.000,0.000][0.000,0.000][0.000,0.000][0.000,0.000][0.000,0.001][0.000,0.015][0.011,0.153][0.134,0.435][0.134,0.435\right]\\ \left[0.000,0.000][0.000,0.000][0.000,0.000][0.000,0.000][0.000,0.001][0.000,0.015][0.000,0.036][0.000,0.036][0.000,0.036\right]\\ \left[0.000,0.000][0.000,0.000][0.000,0.000][0.000,0.000][0.000,0.001][0.000,0.001][0.000,0.001][0.000,0.001][0.000,0.001\right]\\ \left[0.000,0.000][0.000,0.000][0.000,0.000][0.000,0.000][0.000,0.000][0.000,0.000][0.000,0.000][0.000,0.000][0.000,0.000\right]\\ \left[0.000,0.000][0.000,0.000][0.000,0.000][0.000,0.000][0.000,0.000][0.000,0.000][0.000,0.000][0.000,0.000][0.000,0.000\right]\\ \left[0.000,0.000][0.000,0.000][0.000,0.000][0.000,0.000][0.000,0.000][0.000,0.000][0.000,0.000][0.000,0.000][0.000,0.000\right]\\ \left[0.000,0.000][0.000,0.000][0.000,0.000][0.000,0.000][0.000,0.001][0.000,0.015][0.000,0.036][0.000,0.036][0.000,0.036\right]\\ \left[0.000,0.000][0.000,0.000][0.000,0.000][0.000,0.000][0.000,0.001][0.000,0.015][0.000,0.036][0.000,0.036][0.000,0.036\right]\\ \left[0.000,0.000][0.000,0.000][0.000,0.000][0.000,0.000][0.000,0.001][0.000,0.015][0.000,0.036][0.000,0.036][0.000,0.036\right]\\ \left[0.000,0.000][0.000,0.000][0.000,0.000][0.000,0.000][0.000,0.001][0.000,0.001][0.000,0.001][0.000,0.001][0.000,0.001\right]\\ \left[0.000,0.000][0.000,0.000][0.000,0.000][0.000,0.000][0.000,0.000][0.000,0.000][0.000,0.000][0.000,0.000][0.000,0.000\right]\\ \left[0.000,0.000][0.000,0.000][0.000,0.000][0.000,0.000][0.000,0.000][0.000,0.000][0.000,0.000][0.000,0.000][0.000,0.000\right]\\ \left[0.000,0.000][0.000,0.000][0.000,0.000][0.000,0.000][0.000,0.000][0.000,0.000][0.000,0.000][0.000,0.000][0.000,0.000\right]\\ \left[0.000,0.000][0.000,0.000][0.000,0.000][0.000,0.000][0.000,0.001][0.000,0.001][0.000,0.001][0.000,0.001][0.000,0.001\right]\\ \left[0.000,0.000][0.000,0.000][0.000,0.000][0.000,0.000][0.000,0.001][0.000,0.001][0.000,0.001][0.000,0.001][0.000,0.001\right]\\ \left[0.000,0.000][0.000,0.000][0.000,0.000][0.000,0.000][0.000,0.001][0.000,0.001][0.000,0.001][0.000,0.001][0.000,0.001\right]\\ \left[0.000,0.000][0.000,0.000][0.000,0.000][0.000,0.000][0.000,0.001][0.000,0.001][0.000,0.001][0.000,0.001][0.000,0.001\right]\\ \left[0.000,0.000][0.000,0.000][0.000,0.000][0.000,0.000][0.000,0.000][0.000,0.000][0.000,0.000][0.000,0.000][0.000,0.000\right]\\ \left[0.000,0.000][0.000,0.000][0.000,0.000][0.000,0.000][0.000,0.000][0.000,0.000][0.000,0.000][0.000,0.000][0.000,0.000\right]\\ \left[0.000,0.000][0.000,0.000][0.000,0.000][0.000,0.000][0.000,0.000][0.000,0.000][0.000,0.000][0.000,0.000][0.000,0.000\right]\\ \left[0.000,0.000][0.000,0.000][0.000,0.000][0.000,0.000][0.000,0.000][0.000,0.000][0.000,0.000][0.000,0.000][0.000,0.000\right]\\ \left[0.000,0.000][0.000,0.000][0.000,0.000][0.000,0.000][0.000,0.000][0.000,0.000][0.000,0.000][0.000,0.000][0.000,0.000\right]\\ \left[0.000,0.000][0.000,0.000][0.000,0.000][0.000,0.000][0.000,0.000][0.000,0.000][0.000,0.000][0.000,0.000][0.000,0.000\right]\\ \left[0.000,0.000][0.000,0.000][0.000,0.000][0.000,0.000][0.000,0.000][0.000,0.000][0.000,0.000][0.000,0.000][0.000,0.000\right]\\ \left[0.000,0.000][0.000,0.000][0.000,0.000][0.000,0.000][0.000,0.000][0.000,0.000][0.000,0.000][0.000,0.000][0.000,0.000\right]\\ \left[0.000,0.000][0.000,0.000][0.000,0.000][0.000,0.000][0.000,0.000][0.000,0.000][0.000,0.000][0.000,0.000][0.000,0.000\right]\\ \left[0.000,0.000][0.000,0.000][0.000,0.000][0.000,0.000][0.000,0.000][0.000,0.000][0.000,0.000][0.000,0.000][0.000,0.000\right]\\ \left[0.000,0.000][0.000,0.000][0.000,0.000][0.000,0.000][0.000,0.000][0.000,0.000][0.000,0.000][0.000,0.000][0.000,0.000\right]\\ \left[0.000,0.000][0.000,0.000][0.000,0.000][0.000,0.000][0.000,0.000][0.000,0.000][0.000,0.000][0.000,0.000][0.000,0.000\right]\\ \left[0.000,0.000][0.000,0.000][0.000,0.000][0.000,0.000][0.000,0.000][0.000,0.000][0.000,0.000][0.000,0.000][0.000,0.000\right]\\ \left[0.000,0.000][0.000,0.000][0.000,0.000][0.000,0.000][0.000,0.000][0.000,0.000][0.000,0.000][0.000,0.000][0.000,0.000\right]\\ \left[0.000,0.000][0.000,0.000][0.000,0.000][0.000,0.000][0.000,0.000][0.000,0.000][0.000,0.000][0.000,0.000][0.000,0.000\right]\\ \left[0.000,0.000][0.000,0.000][0.000,0.000][0.000,0.000][0.000,0.000][0.000,0.000][0.000,0.000][0.000,0.000][0.000,0.000\right]\\ \left[0.000,0.000][0.000,0.000][0.000,0.000][0.000,0.000][0.000,0.000][0.000,0.000][0.000,0.000][0.000,0.000][0.000,0.000\right]\\ \left[0.000,0.000][0.000,0.000][0.000,0.000][0.000,0.000][0.000,0.000][0.000,0.000][0.000,0.000][0.000,0.000][0.000,0.000\right]\\ \left[0.000,0.000][0.000,0.000][0.000,0.000][0.000,0.000][0.000,0.000][0.000,0.000][0.000,0.000][0.000,0.000][0.000,0.000\right]\\ \left[0.000,0.000][0.000,0.000][0.000,0.000][0.000,0.000][0.000,0.000][0.000,0.000][0.000,0.000][0.000,0.000][0.000,0.000\right]\\ \left[0.000,0.000][0.000,0.000][0.000,0.000][0.000,0.000][0.000,0.000][0.000,0.000][0.000,0.000][0.000,0.000][0.000,0.000\right]\\ \left[0.000,0.000][0.000,0.000][0.000,0.000][0.000,0.000][0.000,0.000][0.000,0.000][0.000,0.000][0.000,0.000][0.000,0.000\right]\\ \left[0.000,0.000][0.000,0.000][0.000,0.000][0.000,0.000][0.000,0.000][0.000,0.000][0.000,0.000][0.000,0.000][0.000,0.000\right]\\ \left[0.000,0.000][0.000,0.000][0.000,0.000][0.000,0.000][0.000,0.000][0.000,0.000][0.000,0.000][0.000,0.000][0.000,0.000\right] \end{array}\right]$$ (35)

In the above calculation, $u_{\underset{\approx }{{PB}_{U}}}(\overset{\approx }{x})$ is the membership degree vector which is the membership degree of control variable *U* belonging to PB.

Similarly, the random membership interval matrix synthesized by the other 24 cloud rules can be obtained. The synthesis method of 25 cloud rules is calculated as follows in Formula (36):

$${{R=R}_{{NB}_{D}\cap {NB}_{DC}\to {PB}_{U}}{\cup R}_{{NB}_{D}\cap {NS}_{DC}\to {PB}_{U}}\cup \ldots \cup R}_{{PB}_{D}\cap {PB}_{DC}\to {NB}_{U}}$$ (36)

Then, *u*_*R*_ can be calculated by following Formula (37):

$${u_{R}=u}_{{R_{{NB}_{D}\cap {NB}_{DC}\to {PB}_{U}}{\cup R}_{{NB}_{D}\cap {NS}_{DC}\to {PB}_{U}}\cup \ldots \cup R}_{{PB}_{D}\cap {PB}_{DC}\to {NB}_{U}}}=u_{R_{{NB}_{D}\cap {NB}_{DC}\to {PB}_{U}}}\vee u_{{NB}_{D}\cap {NS}_{DC}\to {PB}_{U}}\vee \ldots \vee u_{{PB}_{D}\cap {PB}_{DC}\to {NB}_{U}}=$$

$$\left[\begin{array}{c} \left[0.000,0.001][0.000,0.001][0.000,0.001][0.000,0.015][0.011,0.153][0.011,0.154][0.011,0.154][0.324,0.625][1.000,1.000\right]\\ \left[0.000,0.001][0.000,0.001][0.000,0.001][0.000,0.015][0.011,0.153][0.011,0.154][0.011,0.154][0.324,0.625][0.605,0.812\right]\\ \left[0.000,0.001][0.000,0.006][0.000,0.006][0.000,0.015][0.011,0.153][0.134,0.435][0.134,0.435][0.324,0.625][0.605,0.812\right]\\ \left[0.000,0.001][0.000,0.015][0.011,0.153][0.011,0.154][0.011,0.154][0.324,0.625][1.000,1.000][0.324,0.625][0.011,0.154\right]\\ \left[0.000,0.036][0.000,0.036][0.011,0.153][0.011,0.154][0.011,0.154][0.324,0.625][0.605,0.812][0.324,0.625][0.011,0.153\right]\\ \left[0.011,0.153][0.011,0.154][0.011,0.154][0.134,0.435][0.134,0.435][0.324,0.625][0.605,0.812][0.324,0.625][0.011,0.153\right]\\ \left[0.011,0.153][0.011,0.154][0.011,0.154][0.324,0.625][1.000,1.000][0.324,0.625][0.011,0.154][0.011,0.154][0.011,0.153\right]\\ \left[0.000,0.001][0.000,0.001][0.000,0.001][0.000,0.015][0.011,0.153][0.011,0.154][0.011,0.154][0.324,0.625][0.605,0.812\right]\\ \left[0.000,0.001][0.000,0.015][0.000,0.036][0.000,0.036][0.011,0.153][0.324,0.625][0.605,0.812][0.324,0.625][0.134,0.435\right]\\ \left[0.000,0.006][0.000,0.015][0.000,0.036][0.000,0.036][0.011,0.153][0.324,0.625][0.605,0.812][0.324,0.625][0.134,0.435\right]\\ \left[0.000,0.036][0.000,0.036][0.011,0.153][0.011,0.154][0.011,0.154][0.324,0.625][0.605,0.812][0.324,0.625][0.011,0.154\right]\\ \left[0.000,0.036][0.000,0.036][0.011,0.153][0.324,0.625][0.605,0.812][0.324,0.625][0.134,0.435][0.134,0.435][0.011,0.153\right]\\ \left[0.011,0.153][0.134,0.435][0.134,0.435][0.324,0.625][0.605,0.812][0.324,0.625][0.134,0.435][0.134,0.435][0.011,0.153\right]\\ \left[0.011,0.153][0.324,0.625][0.605,0.812][0.324,0.625][0.134,0.435][0.134,0.435][0.011,0.154][0.011,0.154][0.011,0.153\right]\\ \left[0.000,0.001][0.000,0.006][0.000,0.006][0.000,0.015][0.011,0.153][0.134,0.435][0.134,0.435][0.324,0.625][0.605,0.812\right]\\ \left[0.000,0.006][0.000,0.015][0.000,0.036][0.000,0.036][0.011,0.153][0.324,0.625][0.605,0.812][0.324,0.625][0.134,0.435\right]\\ \left[0.000,0.006][0.000,0.015][0.011,0.153][0.134,0.435][0.134,0.435][0.324,0.625][0.605,0.812][0.324,0.625][0.011,0.153\right]\\ \left[0.011,0.153][0.011,0.154][0.011,0.154][0.134,0.435][0.134,0.435][0.324,0.625][0.605,0.812][0.324,0.625][0.011,0.153\right]\\ \left[0.011,0.153][0.134,0.435][0.134,0.435][0.324,0.625][0.605,0.812][0.324,0.625][0.134,0.435][0.134,0.435][0.011,0.153\right]\\ \left[0.011,0.153][0.134,0.435][0.134,0.435][0.324,0.625][0.605,0.812][0.324,0.625][0.011,0.153][0.000,0.036][0.000,0.036\right]\\ \left[0.011,0.153][0.324,0.625][0.605,0.812][0.324,0.625][0.011,0.154][0.011,0.154][0.011,0.153][0.000,0.036][0.000,0.036\right]\\ \left[0.000,0.001][0.000,0.015][0.011,0.153][0.011,0.154][0.011,0.154][0.324,0.625][1.000,1.000][0.324,0.625][0.011,0.154\right]\\ \left[0.000,0.036][0.000,0.036][0.011,0.153][0.011,0.154][0.011,0.154][0.324,0.625][0.605,0.812][0.324,0.625][0.011,0.154\right]\\ \left[0.011,0.153][0.011,0.154][0.011,0.154][0.134,0.435][0.134,0.435][0.324,0.625][0.605,0.812][0.324,0.625][0.011,0.153\right]\\ \left[0.011,0.153][0.011,0.154][0.011,0.154][0.324,0.625][1.000,1.000][0.324,0.625][0.011,0.154][0.011,0.154][0.011,0.153\right]\\ \left[0.011,0.153][0.324,0.625][0.605,0.812][0.324,0.625][0.134,0.435][0.134,0.435][0.011,0.154][0.011,0.154][0.011,0.153\right]\\ \left[0.011,0.154][0.324,0.625][0.605,0.812][0.324,0.625][0.011,0.154][0.011,0.154][0.011,0.153][0.000,0.036][0.000,0.036\right]\\ \left[0.011,0.154][0.324,0.625][1.000,1.000][0.324,0.625][0.011,0.154][0.011,0.154][0.011,0.153][0.000,0.015][0.000,0.001\right]\\ \left[0.000,0.036][0.000,0.036][0.011,0.153][0.011,0.154][0.011,0.154][0.324,0.625][0.605,0.812][0.324,0.625][0.011,0.153\right]\\ \left[0.000,0.036][0.000,0.036][0.011,0.153][0.324,0.625][0.605,0.812][0.324,0.625][0.134,0.435][0.134,0.435][0.011,0.153\right]\\ \left[0.011,0.153][0.134,0.435][0.134,0.435][0.324,0.625][0.605,0.812][0.324,0.625][0.134,0.435][0.134,0.435][0.011,0.153\right]\\ \left[0.011,0.153][0.324,0.625][0.605,0.812][0.324,0.625][0.134,0.435][0.134,0.435][0.011,0.154][0.011,0.154][0.011,0.153\right]\\ \left[0.011,0.153][0.324,0.625][0.605,0.812][0.324,0.625][0.134,0.435][0.134,0.435][0.011,0.153][0.000,0.015][0.000,0.006\right]\\ \left[0.134,0.435][0.324,0.625][0.605,0.812][0.324,0.625][0.011,0.153][0.000,0.036][0.000,0.036][0.000,0.015][0.000,0.006\right]\\ \left[0.605,0.812][0.324,0.625][0.134,0.435][0.134,0.435][0.011,0.153][0.000,0.015][0.000,0.006][0.000,0.006][0.000,0.001\right]\\ \left[0.011,0.153][0.011,0.154][0.011,0.154][0.134,0.435][0.134,0.435][0.324,0.625][0.605,0.812][0.324,0.625][0.011,0.153\right]\\ \left[0.011,0.153][0.134,0.435][0.134,0.435][0.324,0.625][0.605,0.812][0.324,0.625][0.134,0.435][0.134,0.435][0.011,0.153\right]\\ \left[0.011,0.153][0.134,0.435][0.134,0.435][0.324,0.625][0.605,0.812][0.324,0.625][0.011,0.153][0.000,0.036][0.000,0.036\right]\\ \left[0.011,0.154][0.324,0.625][0.605,0.812][0.324,0.625][0.011,0.154][0.011,0.154][0.011,0.153][0.000,0.036][0.000,0.036\right]\\ \left[0.134,0.435][0.324,0.625][0.605,0.812][0.324,0.625][0.011,0.153][0.000,0.036][0.000,0.036][0.000,0.015][0.000,0.006\right]\\ \left[0.134,0.435][0.324,0.625][0.605,0.812][0.324,0.625][0.011,0.153][0.000,0.036][0.000,0.036][0.000,0.015][0.000,0.001\right]\\ \left[0.605,0.812][0.324,0.625][0.011,0.154][0.011,0.154][0.011,0.153][0.000,0.015][0.000,0.001][0.000,0.001][0.000,0.001\right]\\ \left[0.011,0.153][0.011,0.154][0.011,0.154][0.324,0.625][1.000,1.000][0.324,0.625][0.011,0.154][0.011,0.154][0.011,0.153\right]\\ \left[0.011,0.153][0.324,0.625][0.605,0.812][0.324,0.625][0.134,0.435][0.134,0.435][0.011,0.154][0.011,0.154][0.011,0.153\right]\\ \left[0.011,0.153][0.324,0.625][0.605,0.812][0.324,0.625][0.011,0.154][0.011,0.154][0.011,0.153][0.000,0.036][0.000,0.036\right]\\ \left[0.011,0.154][0.324,0.625][1.000,1.000][0.324,0.625][0.011,0.154][0.011,0.154][0.011,0.153][0.000,0.015][0.000,0.001\right]\\ \left[0.605,0.812][0.324,0.625][0.134,0.435][0.134,0.435][0.011,0.153][0.000,0.015][0.000,0.006][0.000,0.006][0.000,0.001\right]\\ \left[0.605,0.812][0.324,0.625][0.011,0.154][0.011,0.154][0.011,0.153][0.000,0.015][0.000,0.001][0.000,0.001][0.000,0.001\right]\\ \left[1.000,1.000][0.324,0.625][0.011,0.154][0.011,0.154][0.011,0.153][0.000,0.015][0.000,0.001][0.000,0.001][0.000,0.001\right] \end{array}\right]$$ (37)

**Clarity of control variable *U*.** When the known condition is an uncertain value and the preconditions rules are not the same as the existing consistency the following similar reasoning is performed.

When the deviation *D = X*_*D*_ and variation of deviation *DC = Y*_*DC*_ are known the cloud reasoning for solving u* = *Z*_*U*_ is as follows in Formula (38):

$$u^{*}=Z_{U}=(X_{D}\cap Y_{DC}){}^{\circ}R$$ (38)

**Fig 5:**
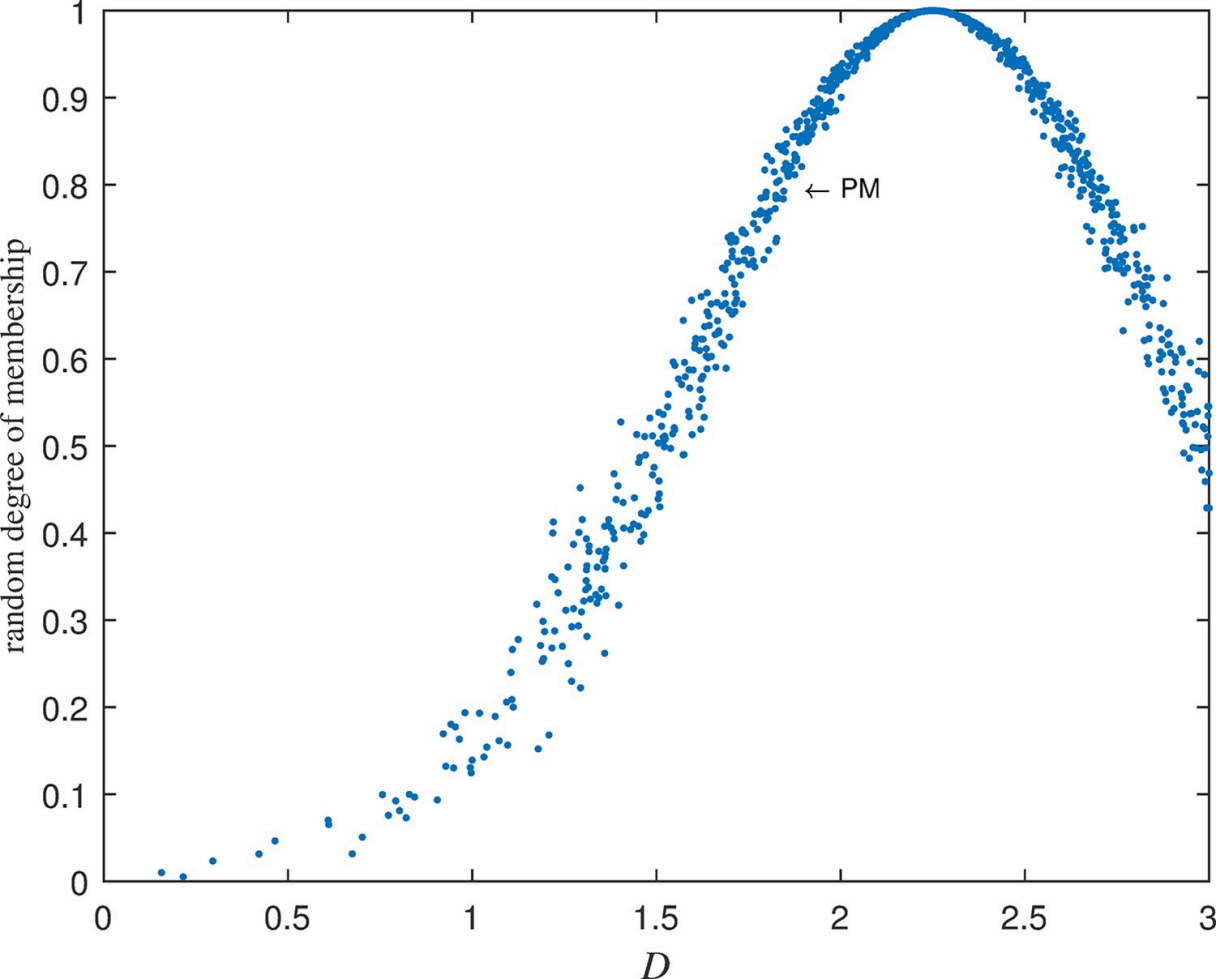
*PM* in deviation *D*.

If the specific language values of observation variables *D* and *DC* and their cloud set presentations are known, how can we solve the unknown values of control variables? It is assumed that the actual cash volume is higher than the safe cash volume. The specific deviation *D* is Positive Middle. It can be abbreviated as PM. It is known that its cloud set is represented as PM_D_~C(2.25,0.637,0.046). Cloud set representation is shown in Fig 5. Membership degree of the cloud subset of seven levels -3, -2, -1, 0, 1, 2, 3 of deviation *D* for PM is calculated to gain the following result in Formula (39):

$$u_{{PM}_{D}}=\{[0.000,0.000][0.000,0.000][0.000,0.000][0.000,0.015][0.043,0.272][0.882,0.949][0.323,0.626]\}$$ (39)

It is assumed that the variation *DC* of the specific deviation is Positive Small (PS)~C(1.5,0.637,0.046) (seeing PS in Fig 2) for schematic diagram. From Table 2 its random membership is denoted as follows in Formula (40):

$$u_{{PS}_{DC}}=\{[0.000,0.000][0.000,0.000][0.000,0.006][0.011,0.154][0.605,0.812][0.605,0.812][0.011,0.154]\}$$ (40)

At this time, the output of the cash flow automatic control system is a vector. The calculation method is as follows in Formula (41):

$$X_{D}\cap Y_{DC}=\left[\begin{array}{c} \left[0.000,0.000\right]\\ \left[0.000,0.000\right]\\ \left[0.000,0.000\right]\\ \left[0.000,0.015\right]\\ \left[0.043,0.272\right]\\ \left[0.882,0.949\right]\\ \left[0.323,0.626\right] \end{array}\right][\left[0.000,0.000][0.000,0.000][0.000,0.006][0.011,0.154][0.605,0.812][0.605,0.812][0.011,0.154\right]]$$

$$=[\begin{array}{c} \left[0.000,0.000][0.000,0.000][0.000,0.000][0.000,0.000][0.000,0.000][0.000,0.000][0.000,0.000\right]\\ \left[0.000,0.000][0.000,0.000][0.000,0.000][0.000,0.000][0.000,0.000][0.000,0.000][0.000,0.000\right]\\ \left[0.000,0.000][0.000,0.000][0.000,0.000][0.000,0.000][0.000,0.000][0.000,0.000][0.000,0.000\right]\\ \left[0.000,0.000][0.000,0.000][0.000,0.006][0.000,0.015][0.000,0.015][0.000,0.015][0.000,0.015\right]\\ \left[0.000,0.000][0.000,0.000][0.000,0.006][0.011,0.154][0.043,0.272][0.043,0.272][0.011,0.154\right]\\ \left[0.000,0.000][0.000,0.000][0.000,0.006][0.011,0.154][0.605,0.812][0.605,0.812][0.011,0.154\right]\\ \left[0.000,0.000][0.000,0.000][0.000,0.006][0.011,0.154][0.323,0.626][0.323,0.626][0.011,0.154\right] \end{array}]$$ (41)

The resulting matrix of above calculation is converted into the column vector to perform the following calculation by bringing into the following Formula (42):

$$u^{*}=(X_{D}\cap Y_{DC}){}^{\circ}R=$$

$${\left[\begin{array}{c} \left[0.000,0.000\right]\\ \left[0.000,0.000\right]\\ \left[0.000,0.000\right]\\ \left[0.000,0.000\right]\\ \left[0.000,0.000\right]\\ \left[0.000,0.000\right]\\ \left[0.000,0.000\right]\\ \left[0.000,0.000\right]\\ \left[0.000,0.000\right]\\ \left[0.000,0.000\right]\\ \left[0.000,0.000\right]\\ \left[0.000,0.000\right]\\ \left[0.000,0.000\right]\\ \left[0.000,0.000\right]\\ \left[0.000,0.000\right]\\ \left[0.000,0.000\right]\\ \left[0.000,0.000\right]\\ \left[0.000,0.000\right]\\ \left[0.000,0.000\right]\\ \left[0.000,0.000\right]\\ \left[0.000,0.000\right]\\ \left[0.000,0.000\right]\\ \left[0.000,0.000\right]\\ \left[0.000,0.006\right]\\ \left[0.000,0.015\right]\\ \left[0.000,0.015\right]\\ \left[0.000,0.015\right]\\ \left[0.000,0.015\right]\\ \left[0.000,0.000\right]\\ \left[0.000,0.000\right]\\ \left[0.000,0.006\right]\\ \left[0.011,0.154\right]\\ \left[0.043,0.272\right]\\ \left[0.043,0.272\right]\\ \left[0.011,0.154\right]\\ \left[0.000,0.000\right]\\ \left[0.000,0.000\right]\\ \left[0.000,0.006\right]\\ \left[0.011,0.154\right]\\ \left[0.605,0.812\right]\\ \left[0.605,0.812\right]\\ \left[0.011,0.154\right]\\ \left[0.000,0.000\right]\\ \left[0.000,0.000\right]\\ \left[0.000,0.006\right]\\ \left[0.011,0.154\right]\\ \left[0.323,0.626\right]\\ \left[0.323,0.626\right]\\ \left[0.011,0.154\right] \end{array}\right]}^{T}{}^{\circ}\left[\begin{array}{c} \left[0.000,0.001][0.000,0.001][0.000,0.001][0.000,0.015][0.011,0.153][0.011,0.154][0.011,0.154][0.324,0.625][1.000,1.000\right]\\ \left[0.000,0.001][0.000,0.001][0.000,0.001][0.000,0.015][0.011,0.153][0.011,0.154][0.011,0.154][0.324,0.625][0.605,0.812\right]\\ \left[0.000,0.001][0.000,0.006][0.000,0.006][0.000,0.015][0.011,0.153][0.134,0.435][0.134,0.435][0.324,0.625][0.605,0.812\right]\\ \left[0.000,0.001][0.000,0.015][0.011,0.153][0.011,0.154][0.011,0.154][0.324,0.625][1.000,1.000][0.324,0.625][0.011,0.154\right]\\ \left[0.000,0.036][0.000,0.036][0.011,0.153][0.011,0.154][0.011,0.154][0.324,0.625][0.605,0.812][0.324,0.625][0.011,0.153\right]\\ \left[0.011,0.153][0.011,0.154][0.011,0.154][0.134,0.435][0.134,0.435][0.324,0.625][0.605,0.812][0.324,0.625][0.011,0.153\right]\\ \left[0.011,0.153][0.011,0.154][0.011,0.154][0.324,0.625][1.000,1.000][0.324,0.625][0.011,0.154][0.011,0.154][0.011,0.153\right]\\ \left[0.000,0.001][0.000,0.001][0.000,0.001][0.000,0.015][0.011,0.153][0.011,0.154][0.011,0.154][0.324,0.625][0.605,0.812\right]\\ \left[0.000,0.001][0.000,0.015][0.000,0.036][0.000,0.036][0.011,0.153][0.324,0.625][0.605,0.812][0.324,0.625][0.134,0.435\right]\\ \left[0.000,0.006][0.000,0.015][0.000,0.036][0.000,0.036][0.011,0.153][0.324,0.625][0.605,0.812][0.324,0.625][0.134,0.435\right]\\ \left[0.000,0.036][0.000,0.036][0.011,0.153][0.011,0.154][0.011,0.154][0.324,0.625][0.605,0.812][0.324,0.625][0.011,0.154\right]\\ \left[0.000,0.036][0.000,0.036][0.011,0.153][0.324,0.625][0.605,0.812][0.324,0.625][0.134,0.435][0.134,0.435][0.011,0.153\right]\\ \left[0.011,0.153][0.134,0.435][0.134,0.435][0.324,0.625][0.605,0.812][0.324,0.625][0.134,0.435][0.134,0.435][0.011,0.153\right]\\ \left[0.011,0.153][0.324,0.625][0.605,0.812][0.324,0.625][0.134,0.435][0.134,0.435][0.011,0.154][0.011,0.154][0.011,0.153\right]\\ \left[0.000,0.001][0.000,0.006][0.000,0.006][0.000,0.015][0.011,0.153][0.134,0.435][0.134,0.435][0.324,0.625][0.605,0.812\right]\\ \left[0.000,0.006][0.000,0.015][0.000,0.036][0.000,0.036][0.011,0.153][0.324,0.625][0.605,0.812][0.324,0.625][0.134,0.435\right]\\ \left[0.000,0.006][0.000,0.015][0.011,0.153][0.134,0.435][0.134,0.435][0.324,0.625][0.605,0.812][0.324,0.625][0.011,0.153\right]\\ \left[0.011,0.153][0.011,0.154][0.011,0.154][0.134,0.435][0.134,0.435][0.324,0.625][0.605,0.812][0.324,0.625][0.011,0.153\right]\\ \left[0.011,0.153][0.134,0.435][0.134,0.435][0.324,0.625][0.605,0.812][0.324,0.625][0.134,0.435][0.134,0.435][0.011,0.153\right]\\ \left[0.011,0.153][0.134,0.435][0.134,0.435][0.324,0.625][0.605,0.812][0.324,0.625][0.011,0.153][0.000,0.036][0.000,0.036\right]\\ \left[0.011,0.153][0.324,0.625][0.605,0.812][0.324,0.625][0.011,0.154][0.011,0.154][0.011,0.153][0.000,0.036][0.000,0.036\right]\\ \left[0.000,0.001][0.000,0.015][0.011,0.153][0.011,0.154][0.011,0.154][0.324,0.625][1.000,1.000][0.324,0.625][0.011,0.154\right]\\ \left[0.000,0.036][0.000,0.036][0.011,0.153][0.011,0.154][0.011,0.154][0.324,0.625][0.605,0.812][0.324,0.625][0.011,0.154\right]\\ \left[0.011,0.153][0.011,0.154][0.011,0.154][0.134,0.435][0.134,0.435][0.324,0.625][0.605,0.812][0.324,0.625][0.011,0.153\right]\\ \left[0.011,0.153][0.011,0.154][0.011,0.154][0.324,0.625][1.000,1.000][0.324,0.625][0.011,0.154][0.011,0.154][0.011,0.153\right]\\ \left[0.011,0.153][0.324,0.625][0.605,0.812][0.324,0.625][0.134,0.435][0.134,0.435][0.011,0.154][0.011,0.154][0.011,0.153\right]\\ \left[0.011,0.154][0.324,0.625][0.605,0.812][0.324,0.625][0.011,0.154][0.011,0.154][0.011,0.153][0.000,0.036][0.000,0.036\right]\\ \left[0.011,0.154][0.324,0.625][1.000,1.000][0.324,0.625][0.011,0.154][0.011,0.154][0.011,0.153][0.000,0.015][0.000,0.001\right]\\ \left[0.000,0.036][0.000,0.036][0.011,0.153][0.011,0.154][0.011,0.154][0.324,0.625][0.605,0.812][0.324,0.625][0.011,0.153\right]\\ \left[0.000,0.036][0.000,0.036][0.011,0.153][0.324,0.625][0.605,0.812][0.324,0.625][0.134,0.435][0.134,0.435][0.011,0.153\right]\\ \left[0.011,0.153][0.134,0.435][0.134,0.435][0.324,0.625][0.605,0.812][0.324,0.625][0.134,0.435][0.134,0.435][0.011,0.153\right]\\ \left[0.011,0.153][0.324,0.625][0.605,0.812][0.324,0.625][0.134,0.435][0.134,0.435][0.011,0.154][0.011,0.154][0.011,0.153\right]\\ \left[0.011,0.153][0.324,0.625][0.605,0.812][0.324,0.625][0.134,0.435][0.134,0.435][0.011,0.153][0.000,0.015][0.000,0.006\right]\\ \left[0.134,0.435][0.324,0.625][0.605,0.812][0.324,0.625][0.011,0.153][0.000,0.036][0.000,0.036][0.000,0.015][0.000,0.006\right]\\ \left[0.605,0.812][0.324,0.625][0.134,0.435][0.134,0.435][0.011,0.153][0.000,0.015][0.000,0.006][0.000,0.006][0.000,0.001\right]\\ \left[0.011,0.153][0.011,0.154][0.011,0.154][0.134,0.435][0.134,0.435][0.324,0.625][0.605,0.812][0.324,0.625][0.011,0.153\right]\\ \left[0.011,0.153][0.134,0.435][0.134,0.435][0.324,0.625][0.605,0.812][0.324,0.625][0.134,0.435][0.134,0.435][0.011,0.153\right]\\ \left[0.011,0.153][0.134,0.435][0.134,0.435][0.324,0.625][0.605,0.812][0.324,0.625][0.011,0.153][0.000,0.036][0.000,0.036\right]\\ \left[0.011,0.154][0.324,0.625][0.605,0.812][0.324,0.625][0.011,0.154][0.011,0.154][0.011,0.153][0.000,0.036][0.000,0.036\right]\\ \left[0.134,0.435][0.324,0.625][0.605,0.812][0.324,0.625][0.011,0.153][0.000,0.036][0.000,0.036][0.000,0.015][0.000,0.006\right]\\ \left[0.134,0.435][0.324,0.625][0.605,0.812][0.324,0.625][0.011,0.153][0.000,0.036][0.000,0.036][0.000,0.015][0.000,0.001\right]\\ \left[0.605,0.812][0.324,0.625][0.011,0.154][0.011,0.154][0.011,0.153][0.000,0.015][0.000,0.001][0.000,0.001][0.000,0.001\right]\\ \left[0.011,0.153][0.011,0.154][0.011,0.154][0.324,0.625][1.000,1.000][0.324,0.625][0.011,0.154][0.011,0.154][0.011,0.153\right]\\ \left[0.011,0.153][0.324,0.625][0.605,0.812][0.324,0.625][0.134,0.435][0.134,0.435][0.011,0.154][0.011,0.154][0.011,0.153\right]\\ \left[0.011,0.153][0.324,0.625][0.605,0.812][0.324,0.625][0.011,0.154][0.011,0.154][0.011,0.153][0.000,0.036][0.000,0.036\right]\\ \left[0.011,0.154][0.324,0.625][1.000,1.000][0.324,0.625][0.011,0.154][0.011,0.154][0.011,0.153][0.000,0.015][0.000,0.001\right]\\ \left[0.605,0.812][0.324,0.625][0.134,0.435][0.134,0.435][0.011,0.153][0.000,0.015][0.000,0.006][0.000,0.006][0.000,0.001\right]\\ \left[0.605,0.812][0.324,0.625][0.011,0.154][0.011,0.154][0.011,0.153][0.000,0.015][0.000,0.001][0.000,0.001][0.000,0.001\right]\\ \left[1.000,1.000][0.324,0.625][0.011,0.154][0.011,0.154][0.011,0.153][0.000,0.015][0.000,0.001][0.000,0.001][0.000,0.001\right] \end{array}\right]=[[0.323,0.626][0.324,0.625][0.605,0.812][0.324,0.625][0.043,0.272][0.043,0.272][0.011,0.154][0.011,0.154][0.011,0.153]]$$ (42)

According to the ideal center of gravity method proposed above, the final result is calculated as follows in Formula (43):

$${ouptput}_{U}=\frac{0.626*(-4)+0.625*(-3)+0.812*(-2)+0.625*(1)+0.043*0+0.043*1+0.011*2+0.011*3+0.011*4}{0.626+0.625+0.812+0.625+0.043+0.043+0.043+0.011+0.011}=-2.285$$ (43)

If deviation *D* is PM then it is clarified according to the "ideal center of gravity method", that is the cash flow can be spent and the control amount of expenditure intensity is: *output*_*U*_ = -2.285.

At the same time other methods can be used to clarity the output of control variable *U*. If the all-maximum -membership method is used, the output is: *output*_*U*_ = -1.3469. If the one-maximum-membership method is used, the output is: *output*_*U*_ = -1.624. If the mean of interval of membership degree method is used, the output is: *output*_*U*_ = -1.6017.

### Intelligent control simulation of cash flow and phase plane analysis

**Fig 6:**
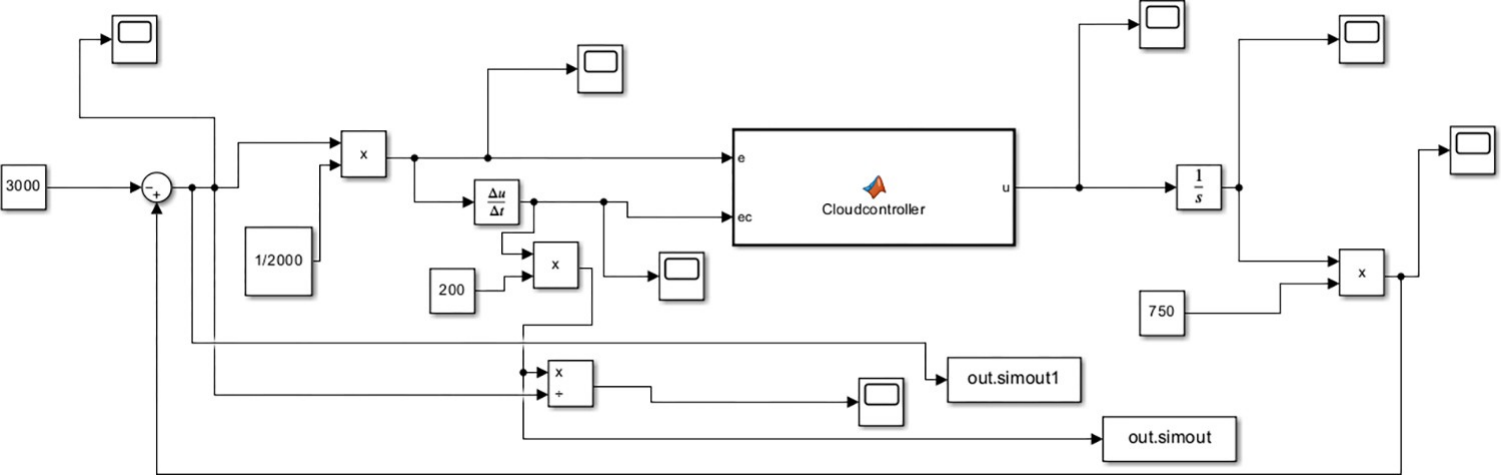
Simulation model based on cloud set.

Assuming a company needs to maintain daily cash flow between -3000 (negative value indicates debt) and 3000 (in US dollars, the same below), for the entire process simulation demonstration of all phase plan divisions, the cash maintenance goal of company is set to a maximum value of 3000, and the initial state is set to -3000. It is easy to know that the deviation range at this time is [-6000, 6000]. The range of deviation variation is limited to [-600, 600]. Initial conditions of intelligent control simulation of cash flow are set as follows: the actual range of deviation *d* is [-6000, 6000], the actual range deviation change *dc* is [-600,600], then mapping factors are set *k*_*d*_ = 1/2000, *k*_*c*_ = 1/200, *k*_*u*_ = 1/750. Use Simulink to establish an intelligent simulation model for the company’s cash flow (seeing Fig 6) and establish a "Cloudcontroller" Matlab Function module based on the method in "Section Cash flow intelligent control decision system based on normal cloud set”.

**Fig 7:**
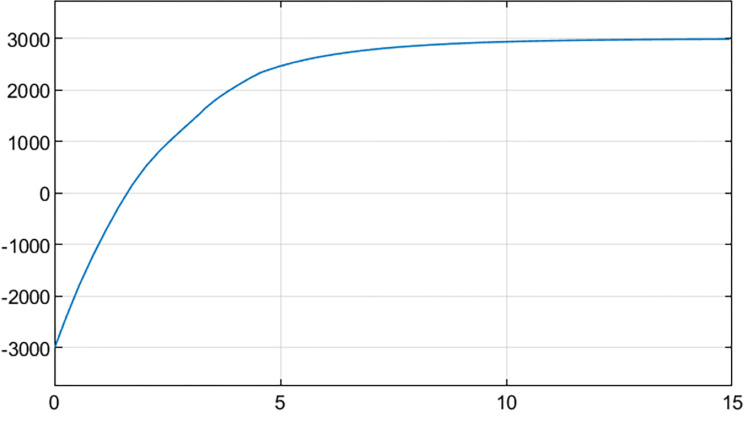
Cash flow change process from -3000 to 3000.

Executing simulation to gain the cash flow change process from -3000 to 3000 in Fig 7. The simulation achieved the goal before 15 steps when output gain is not performed.

**Fig 8:**
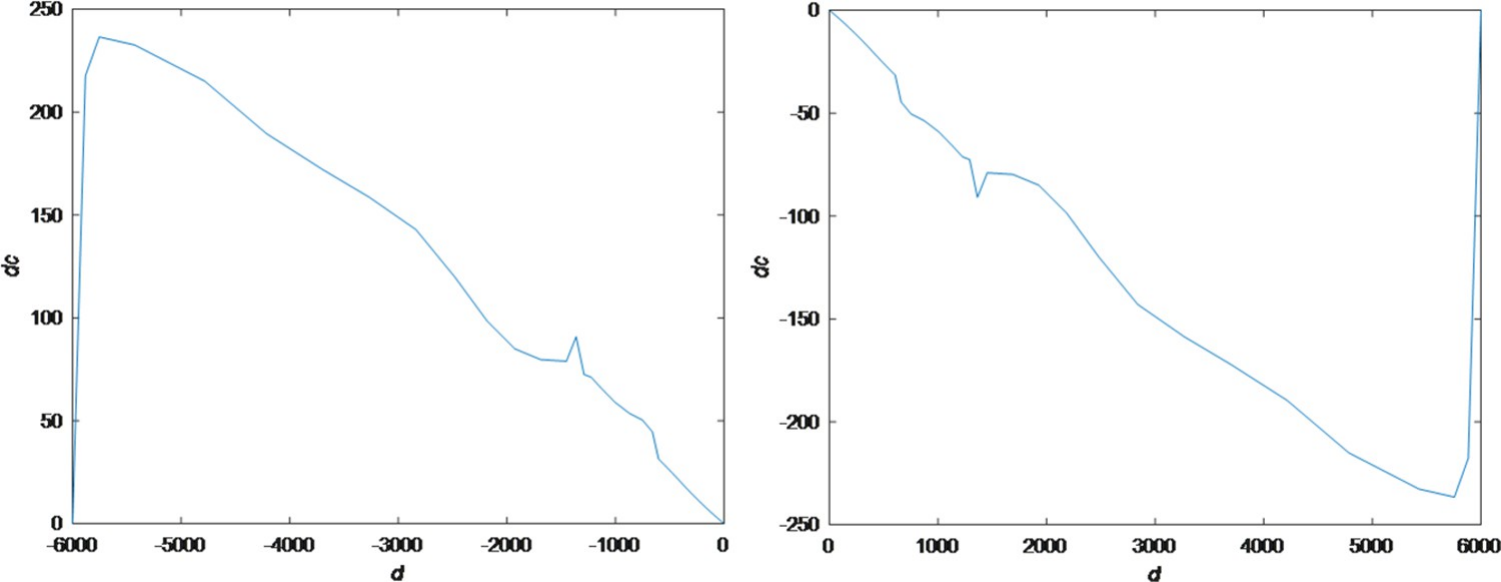
Phase plane diagram for cloud set control. (a) goal=3000, initial value=-3000. (b) goal=-3000, initial value=3000.

At the same time by the “out.simout1” and “out.simout” in Fig 6 the phase plane diagram for cloud set control can be gained in following Fig 8(a). Converting initial conditions: the goal is set to a minimum value of -3000, and the initial state is set to 3000 to gain the phase plane diagram Fig 8(b) which is the inverted symmetry one with the diagram Fig 8(a).

## Comparative analysis with the fuzzy control of cash flow

### The fuzzy control of cash flow

In the following the fuzzy control of cash flow applied in previous research references is provided to compare the difference with cloud set control decision method for cash flow management in this research. Fuzzy membership assignment table of deviation *D* and the variation *DC* of deviation *D* is shown in Table 5. Fuzzy membership assignment table of control variable *U* is shown in Table 6.

**Table 5:** Fuzzy membership assignment table of deviation *D* and the variation *DC* of deviation *D*.

Degree of membership		Level						
		-3	-2	-1	0	1	2	3
**Fuzzy set**	**NB**	1	0.66	0.33	0	0	0	0
	**NS**	0.5	1	0.5	0	0	0	0
	**ZO**	0	0	0.5	1	0.5	0	0
	**PS**	0	0	0	0	0.5	1	0.5
	**PB**	0	0	0	0	0.33	0.66	1

**Table 6:** Fuzzy membership assignment table of control variable *U*.

Degree of membership		Level								
		-4	-3	-2	-1	0	1	2	3	4
**Fuzzy set**	**NB**	1	0.75	0.5	0.25	0	0	0	0	0
	**NS**	0	0.5	1	0.5	0	0	0	0	0
	**ZO**	0	0.25	0.5	0.75	1	0.75	0.5	0.25	0
	**PS**	0	0	0	0	0	0.5	1	0.5	0
	**PB**	0	0	0	0	0	0.25	0.5	0.75	1

**Fig 9:**
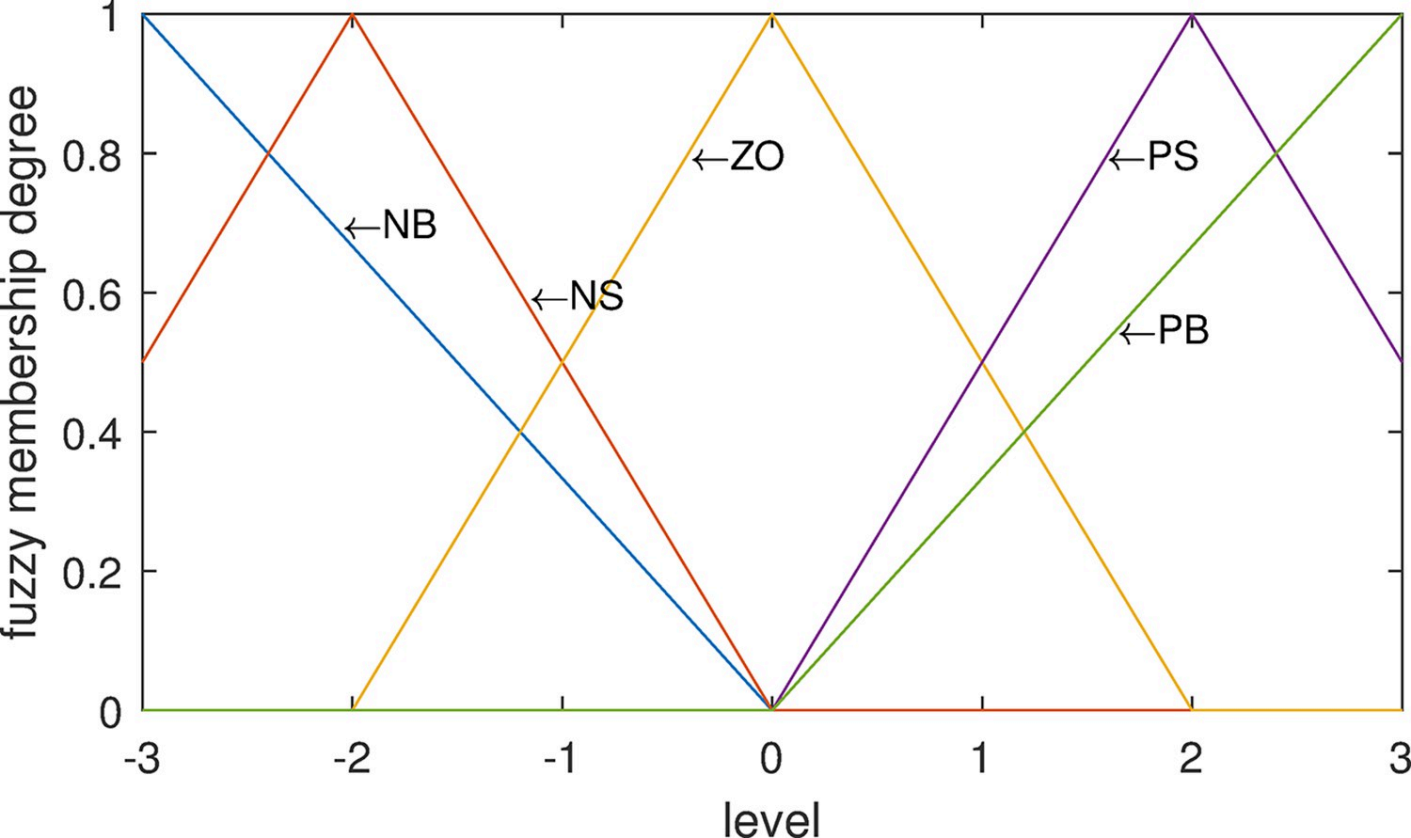
Fuzzy membership function for deviation *D* and the variation *DC* of deviation *D*.

**Fig 10:**
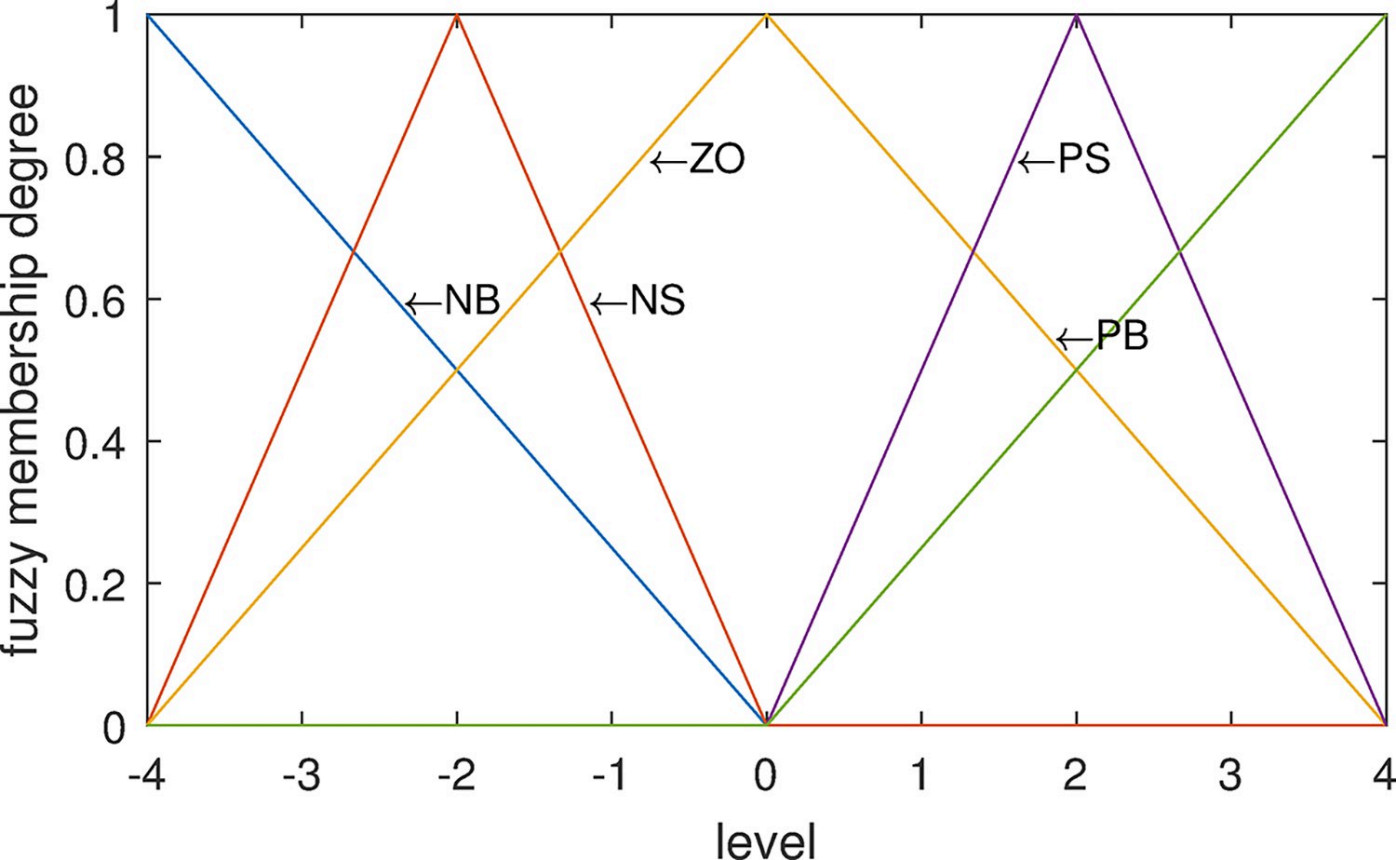
Fuzzy membership function for control variable *U*.

Fuzzy membership functions of deviation *D* and the variation *DC* of deviation *D* are shown in Fig 9. Fuzzy membership functions of control variable *U* are shown in Fig 10.

Fuzzy rules in intelligent control decision of cash flow are the same as the cloud rules in Table 4.

If it is known that its fuzzy set is represented as ${PM}_{D}^{f}=[0,2.5,5]$ then membership degrees of the cloud subsets of seven levels -3, -2, -1, 0, +1, +2, +3 of deviation *D* for PM are calculated to gain the following result in Formula (44):

$$u_{{PM}_{D}}^{f}=\{0,0,0,0,0.4,0.8,0.8\}$$ (44)

It is assumed that the variation *DC* of the specific deviation is Positive Small (PS) = [0, 2, 4], seeing PS in Fig 9 for schematic diagram. In Table 5 its random membership is denoted as follows in Formula (45):

$$u_{{PS}_{DC}}^{f}=\{0,0,0,0,0.5,1,0.5\}$$ (45)

The following fuzzy relation matrix is obtained in Formula (46) through the synthesis of fuzzy reasoning rules:

$$R^{f}=\left[\begin{array}{ccccccccc} 0 & 0 & 0 & 0 & 0 & 0.5000 & 0.5000 & 0.7500 & 1\\ 0 & 0 & 0 & 0 & 0 & 0.5000 & 0.5000 & 0.7500 & 1\\ 0 & 0 & 0 & 0 & 0 & 0.5000 & 0.5000 & 0.5000 & 0.5000\\ 0 & 0 & 0 & 0 & 0 & 0.5000 & 1 & 0.5000 & 0\\ 0 & 0.3300 & 0.5000 & 0.5000 & 0.5000 & 0.5000 & 0.5000 & 0.5000 & 0\\ 0 & 0.5000 & 0.5000 & 0.6600 & 0.6600 & 0.6600 & 1 & 0.5000 & 0\\ 0 & 0.5000 & 0.5000 & 0.7500 & 1 & 0.7500 & 0.5000 & 0.5000 & 0\\ 0 & 0 & 0 & 0 & 0 & 0.5000 & 0.5000 & 0.7500 & 1\\ 0 & 0 & 0 & 0 & 0 & 0.5000 & 1 & 0.6600 & 0.6600\\ 0 & 0 & 0 & 0 & 0 & 0.5000 & 0.5000 & 0.5000 & 0.5000\\ 0 & 0 & 0 & 0 & 0 & 0.5000 & 1 & 0.5000 & 0\\ 0 & 0.3300 & 0.5000 & 0.5000 & 0.5000 & 0.5000 & 0.5000 & 0.5000 & 0\\ 0 & 0.5000 & 0.6600 & 0.7500 & 1 & 0.7500 & 0.6600 & 0.5000 & 0\\ 0 & 0.5000 & 1 & 0.6600 & 0.6600 & 0.6600 & 0.5000 & 0.5000 & 0\\ 0 & 0 & 0 & 0 & 0 & 0.5000 & 0.5000 & 0.5000 & 0.5000\\ 0 & 0 & 0 & 0 & 0 & 0.5000 & 0.5000 & 0.5000 & 0.5000\\ 0 & 0.2500 & 0.5000 & 0.5000 & 0.5000 & 0.5000 & 0.5000 & 0.5000 & 0.3300\\ 0 & 0.2500 & 0.5000 & 0.5000 & 0.5000 & 0.5000 & 0.5000 & 0.5000 & 0\\ 0 & 0.5000 & 0.5000 & 0.5000 & 0.5000 & 0.5000 & 0.5000 & 0.5000 & 0\\ 0 & 0.5000 & 0.5000 & 0.5000 & 0.5000 & 0.5000 & 0.5000 & 0.3300 & 0\\ 0 & 0.5000 & 0.5000 & 0.5000 & 0.5000 & 0.5000 & 0.5000 & 0.3300 & 0\\ 0 & 0 & 0 & 0 & 0 & 0.5000 & 1 & 0.5000 & 0\\ 0 & 0 & 0 & 0 & 0 & 0.5000 & 1 & 0.5000 & 0\\ 0 & 0.2500 & 0.5000 & 0.5000 & 0.5000 & 0.5000 & 0.5000 & 0.5000 & 0\\ 0 & 0.2500 & 0.5000 & 0.7500 & 1 & 0.7500 & 0.5000 & 0.2500 & 0\\ 0 & 0.5000 & 0.5000 & 0.5000 & 0.5000 & 0.5000 & 0.5000 & 0.2500 & 0\\ 0 & 0.5000 & 1 & 0.5000 & 0 & 0 & 0 & 0 & 0\\ 0 & 0.5000 & 1 & 0.5000 & 0 & 0 & 0 & 0 & 0\\ 0 & 0.3300 & 0.5000 & 0.5000 & 0.5000 & 0.5000 & 0.5000 & 0.5000 & 0\\ 0 & 0.3300 & 0.5000 & 0.5000 & 0.5000 & 0.5000 & 0.5000 & 0.5000 & 0\\ 0 & 0.5000 & 0.5000 & 0.5000 & 0.5000 & 0.5000 & 0.5000 & 0.5000 & 0\\ 0 & 0.5000 & 0.5000 & 0.5000 & 0.5000 & 0.5000 & 0.5000 & 0.2500 & 0\\ 0.3300 & 0.5000 & 0.5000 & 0.5000 & 0.5000 & 0.5000 & 0.5000 & 0.2500 & 0\\ 0.5000 & 0.5000 & 0.5000 & 0.5000 & 0 & 0 & 0 & 0 & 0\\ 0.5000 & 0.5000 & 0.5000 & 0.5000 & 0 & 0 & 0 & 0 & 0\\ 0 & 0.5000 & 0.5000 & 0.6600 & 0.6600 & 0.6600 & 1 & 0.5000 & 0\\ 0 & 0.5000 & 0.6600 & 0.7500 & 1 & 0.7500 & 0.6600 & 0.5000 & 0\\ 0 & 0.5000 & 0.5000 & 0.5000 & 0.5000 & 0.5000 & 0.5000 & 0.3300 & 0\\ 0 & 0.5000 & 1 & 0.5000 & 0 & 0 & 0 & 0 & 0\\ 0.5000 & 0.5000 & 0.5000 & 0.5000 & 0 & 0 & 0 & 0 & 0\\ 0.6600 & 0.6600 & 1 & 0.5000 & 0 & 0 & 0 & 0 & 0\\ 1 & 0.7500 & 0.5000 & 0.5000 & 0 & 0 & 0 & 0 & 0\\ 0 & 0.5000 & 0.5000 & 0.7500 & 1 & 0.7500 & 0.5000 & 0.5000 & 0\\ 0 & 0.5000 & 1 & 0.6600 & 0.6600 & 0.6600 & 0.5000 & 0.5000 & 0\\ 0 & 0.5000 & 0.5000 & 0.5000 & 0.5000 & 0.5000 & 0.5000 & 0.3300 & 0\\ 0 & 0.5000 & 1 & 0.5000 & 0 & 0 & 0 & 0 & 0\\ 0.5000 & 0.5000 & 0.5000 & 0.5000 & 0 & 0 & 0 & 0 & 0\\ 1 & 0.7500 & 0.5000 & 0.5000 & 0 & 0 & 0 & 0 & 0\\ 1 & 0.7500 & 0.5000 & 0.5000 & 0 & 0 & 0 & 0 & 0 \end{array}\right]$$ (46)

Then, the result of fuzzy control is resolved by the following Formula (47):

$$u_{U}^{f}=(u_{{PM}_{D}}^{f}\wedge u_{{PS}_{DC}}^{f}){}^{\circ}R^{f}=[0.8,0.75,0.8,0.5,0.4,0.4,0.4,0.25,0]$$ (47)

If the classic maximum fuzzy membership degree method is used the fuzzy control variable *U*^*f*^ may be calculated by the following Formula (48):

$$U^{f}=(-4*0.8+(-2)*0.8)/2=-2.4$$ (48)

### Intelligent control simulation of cash flow and phase plane analysis based on fuzzy controller

**Fig 11:**
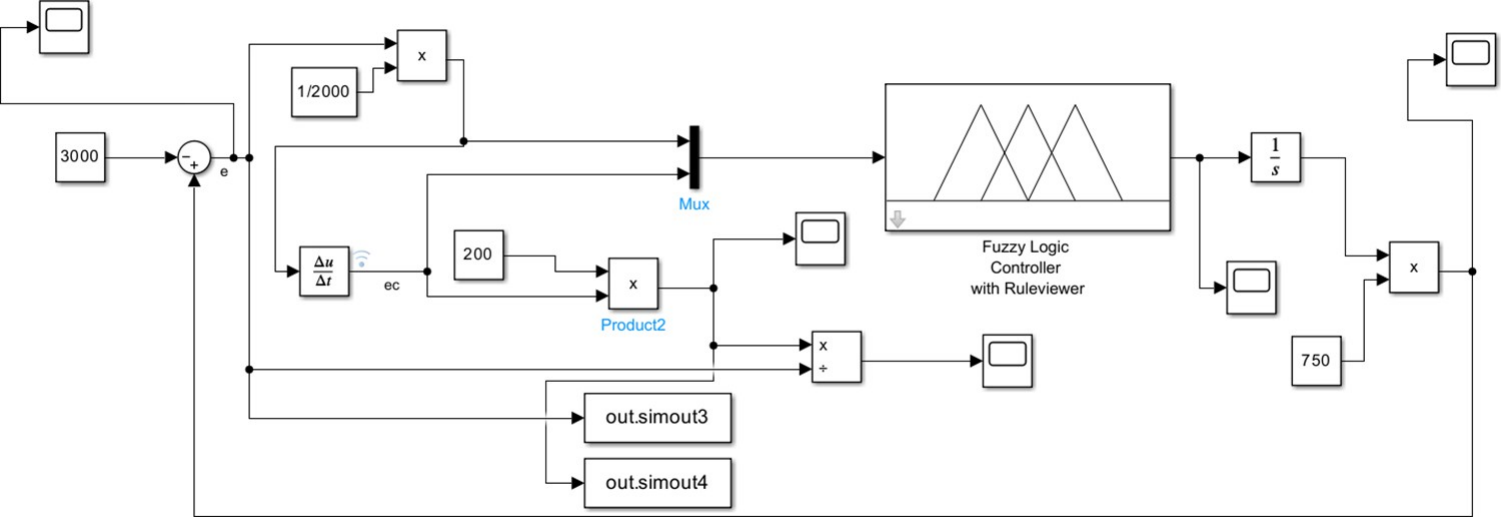
Simulation model based fuzzy set.

Under the same conditions, simulation model (seeing Fig 11) using fuzzy logic is created to execute the simulation process. This model named “Fuzzy Logic Controller with Ruleviewer” is created by Fuzzy Logic Designer in Matlab using classic fuzzy reasoning based on Mamdani method.

**Fig 12:**
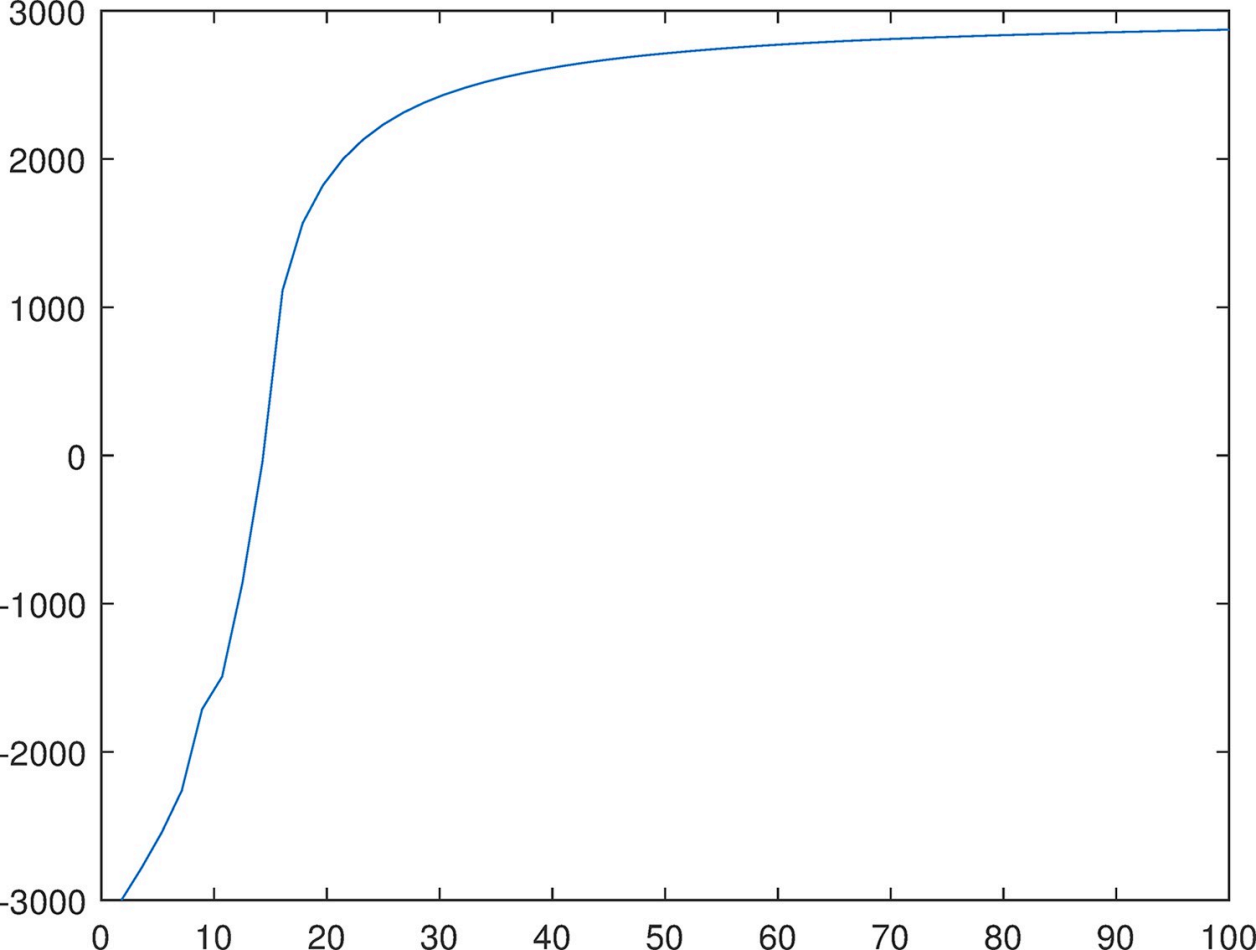
Cash flow change process from -3000 to 3000 in fuzzy control simulation.

Cash flow change process from -3000 to 3000 in fuzzy control simulation is shown in Fig 12. The simulation closely achieved the goal after 100 steps when output gain is not performed.

**Fig 13:**
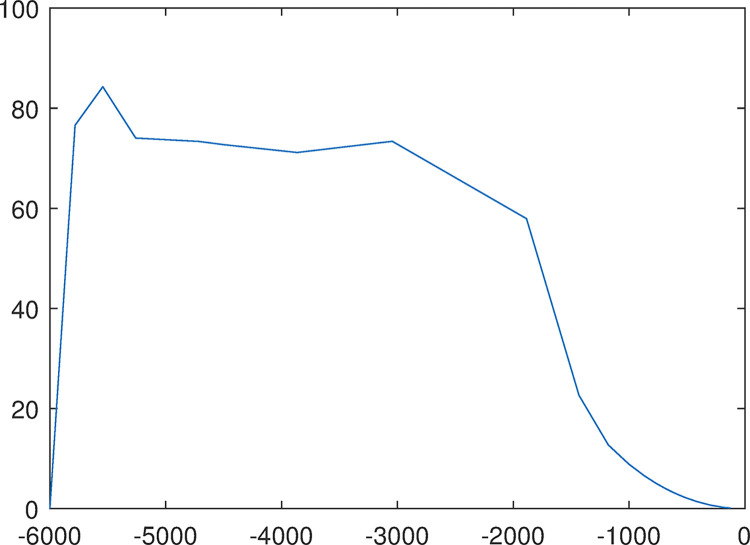
Phase plane diagram for fuzzy set.

Phase plane diagram for fuzzy set is shown in Fig 13.

### Comparative analysis

From the comparison of the method proposed by this paper with fuzzy control of cash flow, the following advantages of intelligent control of cash flow based on cloud set can be found:

1. The fuzziness and randomness are integrated into one organic whole. The randomness can be fully expressed and integrated with fuzziness by the hyper-entropy *He* in intelligent control of cash flow based on cloud set. The membership functions are random curves in Figs 2–5 for intelligent control of cash flow based on cloud set but not the fixed curve in Figs 9 and 10 in fuzzy linear programming. This random curve of membership is more objective and reasonable than the fixed curve because it just expresses the way the human brain makes the decision and judgement.
2. The uncertainty is objectively and effectively expressed and brought into the operation process. Intelligent control of cash flow based on cloud set in Fig 7 can gain the optimal value which is faster than the value of fuzzy control in Fig 12 of cash flow. This indicates that randomness increases the range of conditional selection of variables. Fuzzy control of cash flow without randomness ignores this point, however intelligent control of cash flow based on cloud set fully expresses the uncertainty of decision. By the comparison of phase plane diagram between cloud set in Fig 8 and fuzzy set in Fig 13, it can be seen the cloud set controller provides the faster change.
3. Options of decisions methods can be selected as required. If you do not care about the randomness and reflect its influence in your decisions then you may use the fuzzy control. However, this method is often criticized for being too subjective. If randomness has to be considered for your decision, then intelligent control based on cloud set is a good alternative.

## Conclusion

In the company the cash management has an important role in the benign development of company. Cash flow automation intelligent control decision can improve the efficiency of cash flow management. The automation intelligent control decision method based on cloud set can overcome the shortcoming of lacking randomness. The cloud logic and its reasoning method based on cloud set can be effectively applied in the approximate reasoning for cash flow intelligent control decision. Different from traditional fuzzy approximate reasoning, cloud reasoning turns the random membership degree into an interval value to participate in the calculation, which overcomes the objectivity of the single membership of traditional fuzzy mathematics. This method can be applied to automatic intelligent control decision in other economic and social fields.

The limitations of the proposed model are as follows: (1) The research in this paper is only the automation of a single link of control of cash management. It may require the combination of system theory and methods such as system dynamics or parallel system theory to establish a comprehensive intelligent control system. In such a comprehensive system, there are a larger number of variables that require the usage of cloud sets to handle and express fuzziness and randomness. This will further test the stability of the method. (2) This method only involves the size of cash flow as a considered factor. This study did not specifically consider other external factors that affect cash flow. These external factors may limit the control of cash flow and limit the increase or decrease of cash flow. These factors affect the company’s ability to control cash flow and even make it impossible to control cash flow, such as extreme situations of cash flow disruptions. It is necessary to apply the fuzziness and randomness of cloud models to comprehensively express the influence of external factors so that the system has a consistent variable model expression foundation. This will involve more new operations on cloud set that integrate fuzziness and randomness. (3) Rules that are not adaptive may affect the system’s response to environmental changes. Once the control rules are determined, they cannot adapt to changes in the environment and require manual adjustment. Adaptive rules need to be studied for this method to overcome this weakness. How to establish adaptive rules based on cloud set integrating fuzziness and randomness is a new task.

Potential practical implementation challenges of intelligent control of cash flow based on cloud set method are as follows: (1) Data availability. Data needs to be entered into the control system in a timely and accurate manner in order to improve the application effectiveness of the method. Collecting cash flow management information through real-time information systems can improve the availability of information. At the same time, it is necessary to combine other data factors to predict cash flow, so the availability of other data information that affects cash flow is also very important. (2) Model calibration. In the process of using real data for model calibration, both fuzziness and randomness attributes need to be addressed simultaneously. Some key model calibration processes face new problems and require new methods, including setting the number of levels of *D*, *DC*, and *U* in the model, selecting the suitable sizes for expected *Ex*, entropy *En*, and super entropy *He*, and selecting clear methods for *U*’s cloud set output, etc. (3) User interface design. A natural language-based user interface will make it easier for ordinary users to operate the method with intelligent control. It is difficult to accurately demonstrate the randomness and fuzziness of the model to ordinary users through a graphical user interface. The methods implemented based on Simulink and MATLAB need to be further transformed into independent user-friendly interface systems both for ordinary and professional users.

The future perspectives of relative studies are as follows:(1) Automation of control has been expanded to cover the whole chain of cash management in future studies. (2) The control model involving multi-control variables based on cloud set should be studied. (3) It can be further studied that combining the external factors that affect the cash flow will make this method more objective and practical. These external factors will further complement the comprehensive approach of the intelligent control method based on cloud set for automatic management of cash flow. More factors can be internalized into cloud rules to participate in approximate reasoning and improve the reliability of applications. These factors possibly include current assets and fixed assets, short-term loans and long-term liabilities, main business income and other income, production costs, sales costs, management expenses, sales expenses, operating profit and net profit, intellectual property status, brand value, sales status and its predicted value, enterprise credit rating, and operating status, etc. They can be divided into direct or indirect factors. The system can use nested and hierarchical systems of rules to implement and express the impact of these rules on cash flow. As cloud set integrating fuzziness and randomness can be applied in the intelligent prediction, control, and decision of cash flow considering these factors, the system’s capabilities to handle uncertainty will be enhanced. The more comprehensive the influencing factors are considered, the more accurate the prediction of cash is, and the clearer the control of cash is.
